# Metagenomic Insights Into Competition Between Denitrification and Dissimilatory Nitrate Reduction to Ammonia Within One-Stage and Two-Stage Partial-Nitritation Anammox Bioreactor Configurations

**DOI:** 10.3389/fmicb.2022.825104

**Published:** 2022-04-25

**Authors:** Samuel J. Bryson, Kristopher A. Hunt, David A. Stahl, Mari-Karoliina H. Winkler

**Affiliations:** Department of Civil and Environmental Engineering, University of Washington, Seattle, WA, United States

**Keywords:** metagenome, denitrification, DNRA, PNA, Anammox (anaerobic ammonium oxidation)

## Abstract

Anaerobic ammonia oxidizing bacteria (Anammox) are implemented in high-efficiency wastewater treatment systems operated in two general configurations; one-stage systems combine aerobic ammonia oxidizing bacteria (AOB) and Anammox within a single aerated reactor, whereas two-stage configurations separate these processes into discrete tanks. Within both configurations heterotrophic populations that perform denitrification or dissimilatory nitrate reduction to ammonia (DNRA) compete for carbon and nitrate or nitrite and can impact reactor performance because DNRA retains nitrogen in the system. Therefore, it is important to understand how selective pressures imposed by one-stage and two-stage reactor configurations impact the microbial community structure and associated nitrogen transforming functions. We performed 16S rRNA gene and metagenomic sequencing on different biomass fractions (granules, flocs, and suspended biomass) sampled from two facilities treating sludge dewatering centrate: a one-stage treatment facility (Chambers Creek, Tacoma, WA) and a two-stage system (Rotterdam, Netherlands). Similar microbial populations were identified across the different samples, but relative abundances differed between reactor configurations and biomass sources. Analysis of metagenome assembled genomes (MAGs) indicated different lifestyles for abundant heterotrophic populations. *Acidobacteria*, *Bacteroidetes*, and *Chloroflexi* MAGs had varying capacity for DNRA and denitrification. *Acidobacteria* MAGs possessed high numbers of glycosyl hydrolases and glycosyl transferases indicating a role in biomass degradation. *Ignavibacteria* and *Phycosphaerae* MAGs contributed to the greater relative abundance of DNRA associated *nrf* genes in the two-stage granules and contained genomic features suggesting a preference for an anoxic or microoxic niche. In the one-stage granules a MAG assigned to Burkholderiales accounted for much of the abundant denitrification genes and had genomic features, including the potential for autotrophic denitrification using reduced sulfur, that indicate an ability to adapt its physiology to varying redox conditions. Overall, the competition for carbon substrates between denitrifying and DNRA performing heterotrophs may be impacted by configuration specific selective pressures. In one-stage systems oxygen availability in the bulk liquid and the oxygen gradient within granules would provide a greater niche space for heterotrophic populations capable of utilizing both oxygen and nitrate or nitrite as terminal electron acceptors, compared to two-stage systems where a homogeneous anoxic environment would favor heterotrophic populations primarily adapted to anaerobic metabolism.

## Introduction

The discovery of anaerobic ammonia oxidizing bacteria (Anammox) has revolutionized our understanding of nitrogen cycling (Strous et al., 1999) and also presented an opportunity for the development of energy efficient wastewater treatment systems (WWTS) (Kartal et al., 2010; Lackner et al., 2014). Anammox bacteria form a monophyletic lineage within the phylum *Planctomycetes* that can couple the reduction of nitrite with the oxidation of ammonia to form dinitrogen gas (van de Graaf et al., 1997; Kartal et al., 2011). Compared to conventional wastewater treatment systems (WWTS), Anammox based systems offer 60% reduction in aeration energy, do not require organic carbon, and produce significantly less sludge (Jetten et al., 1997; Lackner et al., 2014; Cho et al., 2019).

About 75% of current Anammox implementations treat ammonia laden side-stream wastewater generated during the dewatering process of sludge from an anaerobic digestor within municipal WWTS (Lackner et al., 2014) with the remainder of implementations treating ammonia rich wastewater from other sources. Anammox bacteria must be partnered with aerobic ammonia oxidizing bacteria (AOB) that provide Anammox with their electron acceptor, nitrite. There are two distinct reactor configurations that promote this partnership (van der Star et al., 2007): the one-stage or single reactor partial-nitritation Anammox (PNA) process in which AOB and Anammox concomitantly grow in one single reactor (Third et al., 2001; Sliekers et al., 2002; Joss et al., 2009) and the two-stage PNA process in which sequential aerated and anoxic reactors separate the aerobic ammonium oxidation to nitrite and Anammox processes (van Dongen et al., 2001; van der Star et al., 2007). While much research has focused on the performance of one-stage and two-stage Anammox reactor configurations, the impact of different reactor configurations on their microbial community structure and function is less studied.

The application of omics tools (e.g., metagenomics and metatranscriptomics) to investigate PNA reactor microbial communities has primarily focused on understanding the biology of Anammox bacteria (Strous et al., 2006; Gori et al., 2011; Hu et al., 2012; Speth et al., 2012; Bhattacharjee et al., 2017), but a growing number of studies have examined the diversity of microorganisms found in PNA reactor biomass. These accessory populations may have important functional roles in biofilm formation, granule stability, and may be involved in syntrophic or competitive interactions that impact the overall reactor performance and operational stability. Studies utilizing 16S rRNA gene amplicon sequencing to analyze biomass sampled from full-scale one-stage and two-stage PNA systems (Gonzalez-Gil et al., 2015; Gonzalez-Martinez et al., 2015a,b) have consistently identified abundant OTUs assigned to *Bacteroidetes*, *Proteobacteria*, *Planctomycetes*, *Ignavibacteria*, *Chloroflexi*, *Chlorobi*, *Acidobacteria*, and *Verrucomicrobia* phyla, suggesting a consistent niche for heterotrophic microorganisms in PNA reactors.

Several studies have focused on the potential nitrogen transforming roles of these organisms in PNA reactor microbial communities. Analysis of metagenome assembled genomes (MAGs) recovered from a one-stage PNA reactor at a plant treating potato-processing wastewater identified incomplete denitrification pathways among many of the heterotrophic populations (Speth et al., 2016), indicating their potential ecological role in a nitrite loop, through which nitrate produced by Anammox bacteria (Kartal et al., 2011) would be converted back to nitrite that can then be converted along with ammonia to nitrogen gas (Winkler et al., 2012b). In another study, analysis of MAGs recovered from flocs, biofilm, and granules sampled from three different one-stage Anammox reactor configurations indicated a high abundance of nitrate reductase (*narG*) genes in all samples (Guo et al., 2016), further supporting the hypothesis of a denitrifier mediated nitrite loop occurring within these systems. Analyses of MAGs recovered from lab scale Anammox reactors have identified abundant heterotrophic populations with the genomic potential for cross-feeding interactions, including exopolysaccharide production by *Chloroflexi* populations and degradation of proteins by *Chlorobi* populations (Zhao et al., 2018). Furthermore, a metatranscriptomic analysis indicated the expression of extracellular peptidases by *Chlorobi* populations suggesting the hypothesis that the degradation of proteins potentially produced by Anammox bacteria serves as a carbon source for the reduction of nitrate by heterotrophic community members (Lawson et al., 2017).

Although cross-feeding between Anammox and denitrifying heterotrophs may sustain a nitrite loop or even enhance a PNA reactor’s overall nitrogen removal performance through conversion of nitrate to dinitrogen gas, heterotrophs that perform dissimilatory nitrate reduction to ammonia (DNRA), which utilizes the *nrf* mediated one step reduction of nitrite to ammonia (Einsle et al., 2002) may disrupt the nitrite loop and increase overall aeration demands for a PNA system. The presence of either *nar* or *nap* nitrate reductase genes by sympatric denitrifying and DNRA performing heterotrophs would foster competition for nitrate produced by Anammox and for available carbon sources, such as soluble organic compounds produced by the microbial community or supplied to the reactor. However, incomplete nitrogen transformation pathways are common among heterotrophs in WWTS (Gao et al., 2019), and the co-occurrence of *nar* or *nap* nitrate reductase genes in addition to *nir* or *nrf* nitrite reductases is not consistent (Speth et al., 2016). Accordingly, competition for nitrite and organic carbon between heterotrophs and Anammox may also impact nitrogen removal due to the capacity of Anammox bacteria to perform DNRA by oxidizing organic acids such as acetate and propionate to carbon dioxide (Castro-Barros et al., 2017). While Anammox bacteria are capable of oxidizing organic acids, assimilation of carbon has not been detected, suggesting that Anammox maintains autotrophic growth, which is an unsolved puzzle (Güven et al., 2005; Kartal et al., 2007a,b). Overall, the competition for nitrate/nitrite and carbon sources between autotrophic or organotrophic Anammox metabolism and heterotrophic denitrification or DNRA metabolism, may have net positive or negative impacts on reactor performance. For example, a time series metagenomic analysis of a lab-scale PNA reactor found multiple taxa capable of DNRA which ultimately outcompeted Anammox and led to reduced nitrogen removal performance in the reactor (Keren et al., 2020). Alternatively, a PNA reactor fed with acetate and ammonium at a low carbon to nitrogen ration (0.5 gCOD/gN) and controlled at a low oxygen concentration (1.5 mg O_2_/L) was able to maintain stable nitrogen removal performance (Winkler et al., 2012b).

Although the presence of heterotrophic populations capable of DNRA, indicated by the presence of *nrf* genes, has been consistently reported in metagenomic studies of PNA reactors (Guo et al., 2016; Speth et al., 2016; Lawson et al., 2017; Wang et al., 2019), understanding the selective pressures that govern competition between DNRA and denitrification remains an active area of research for both engineered and natural ecosystems. These competing metabolisms have been investigated in terms of (i) the reaction energetics and growth yield, (ii) the interaction between reaction stoichiometries and carbon and nitrate substrate availability, and (iii) kinetic parameters. For example, the expected free energy gained from complete denitrification of nitrate to dinitrogen (ΔG°’ = –2.670 kJ per mol glucose) is greater than for DNRA (ΔG°’ = –1.870 kJ per mol glucose), however, experiments have demonstrated that the growth yield from denitrification can be far lower than expected, even lower than for DNRA (Strohm et al., 2007), suggesting that the factors governing the competition are more complex than can be predicted by theoretical reaction yields alone. Furthermore, analyses of natural soil and marine microbial communities have examined how factors including the carbon to nitrate ratio, the nitrite to nitrate ratio, as well as the impacts of pH, temperature, and carbon source influence which of the two processes dominates (Rütting et al., 2011; Kraft et al., 2014; Pandey et al., 2020). Chemostat studies have also demonstrated the influence of the type of carbon source on the competitive outcome, indicating that fermentable substrates may provide even greater selective pressure for DNRA than the carbon to nitrate ratio alone (van den Berg et al., 2016, 2017).

Overall, it seems that the competition between heterotrophic denitrification and heterotrophic DNRA is more complex than one that can be reduced to a single controlling variable. Mathematical models utilizing resource-ratio theory suggest that the impact of carbon to nitrate ratio on competition between heterotrophic denitrification and heterotrophic DNRA is affected by substrate concentration and dilution rate, as well as the affinity and maximum growth rate parameters that may in turn be influenced by environmental conditions such as pH and temperature (Jia et al., 2020). Thus, it may be more important to examine the genomic traits and ecological roles or life strategies of the specific heterotrophs performing denitrification, DNRA, or both, in order to assess how environmental factors influence specific competitive outcomes. Within PNA systems, this competition is further complicated by competition for carbon sources between heterotrophs and Anammox. Anammox bacteria have been shown to outcompete heterotrophs for organic acids and nitrite supplied in the media (Kartal et al., 2007b; Winkler et al., 2012a,b), primarily reducing nitrate or nitrite to generate substrates for anaerobic ammonia oxidation. However, heterotrophic populations may outcompete Anammox bacteria when the carbon to nitrogen ratio (gCOD/gN) is greater than one (Güven et al., 2005).

Given the nuanced nature of the competition between denitrification and DNRA, and its potential impact on PNA reactor performance, it is important to examine the heterotrophic microbial communities found in the different types of PNA reactor configurations—to identify which populations perform denitrification or DNRA and further characterize the genomic features that contribute toward their success. The primary distinguishing factor differentiating one-stage and two-stage PNA reactors is the presence of oxygen in the bulk liquid environment and this likely represents a significant factor in determining the available niches and the types of heterotrophic populations that are selected for. To improve our understanding of how reactor configuration impacts the microbial communities within PNA systems we characterized the microbial communities of a one-stage (van Dongen et al., 2001; Abma et al., 2007; van der Star et al., 2007) and a two-stage system (Klein et al., 2018) treating side-stream sludge anaerobic digester centrate. We performed 16S rRNA gene sequencing and shotgun metagenomic analyses to identify the microbial populations present in each system and assess structural and functional differences between the communities. Furthermore, we compared genomic features of the abundant heterotrophic populations to identify factors that may contribute to selection for DNRA or denitrification, and thus infer how operational strategies of the two PNA configurations impact microbial community composition and net reactor performance.

## Materials and Methods

### Sample Collection and DNA Prep

Biomass and mixed liquor samples were collected from a one-stage PNA bioreactor at Chamber’s Creek Regional Wastewater Treatment Plant, Tacoma, WA, United States (Klein et al., 2018), and a two-stage reactor in Olbergen, Rotterdam, the Netherlands (van Dongen et al., 2001; Abma et al., 2007; van der Star et al., 2007). Material was separated by centrifugation at 5,000 rcf in a 50 mL tube resulting in three distinct layers: granules, flocs, and suspended fractions from the one-stage reactor, and only granules and suspended material from the two-stage system. The different fractions were separated and transferred to 2 mL tubes then collected by pelleting at 20,000 rcf. DNA extraction was performed using the Qiagen DNEasy Power Biofilm Kit (Qiagen, Germany) on the pelleted biomass. DNA was quantified using a NanoDrop 2000 (Thermo Fisher Scientific, Wilmington, Germany).

### 16S rRNA Gene Library Sequencing and Analysis

Aliquots of DNA extract were diluted to ∼25 ng μL^–1^ and ∼20 μL was sent to MRDNA labs (Texas, United States) for amplification of the v4-v5 region of the 16S rRNA gene using primers 515F-Y and 926R (Parada et al., 2016; Walters et al., 2016). Amplicon libraries were sequenced using the MiSeq platform producing 2 × 300 bp paired-end reads. Forward and reverse amplicon libraries were processed (merged and filtered) using the Usearch Pipeline (Edgar, 2010). Zero-radius operational taxonomic units (OTUs) were selected using the Unoise algorithm (Edgar, 2016b) with a minimum of five sequences across all five samples. OTU taxonomy was assigned using the syntax command (Edgar, 2016a) and the RDP 16S rRNA training set v16 (Maidak et al., 1997).

### Metagenome Sequencing, Assembly, and Analysis

DNA aliquots of each biomass fraction from the two types of reactors (five in total) were sequenced using both short-read (Illumina) and long-read (Oxford Nanopore) platforms. For short-read sequencing, DNA was sent to BGI America for library preparation and sequencing on the Illumina HiSeq X-Ten platform producing 2 × 150 bp paired-end reads. Oxford Nanopore MinION (Jain et al., 2016) sequencing was performed on each of the same five DNA extractions. Libraries were prepared using the Rapid Sequencing Kit (SQK-RAD004) and samples from each reactor were run on the same flow cell (two flow cells total) using the Flow Cell Wash Kit (EXP-WSH004) between runs. Base calling was performed locally using Albacore (Oxford Nanopore Technologies) and the resulting fastq files were combined for each reactor configuration and then corrected using the Canu pipeline (Koren et al., 2017).

All short-read and corrected long-read sequence libraries were uploaded to the DOE KBase platform (Arkin et al., 2018) for assembly and binning. Short-read libraries were quality filtered and trimmed using Trimmomatic (Bolger et al., 2014) prior to assembly. The one-stage and two-stage sequence library sets were independently combined and then assembled using different algorithms and compared using QUAST (Gurevich et al., 2013) before selecting one assembly for final analysis. Short-read libraries were assembled with metaSPADEs (Nurk et al., 2017), IDBA-UB (Peng et al., 2012), and MegaHIT (Li et al., 2015), with metaSPADEs producing the longest and most contiguous assembly. Corrected long-reads were co assembled with short-reads using hybridSPAdes (Antipov et al., 2016). Both the metaSPADEs and hybridSPADES assemblies were binned using MaxBin2 (Wu et al., 2016) and bins were analyzed with CheckM (Parks et al., 2015). Results from the metaSPADEs and hybridSPADES assemblies are presented in Supplementary Table 1. The hybridSPADEs assembly was selected for further analysis. Gene calling for scaffolds was performed with PRODIGAL (Hyatt et al., 2010) and predicted coding sequences (CDS) were annotated using the EggNOG (Huerta-Cepas et al., 2019) database and EggNOGmapper.py (Huerta-Cepas et al., 2017) in diamond (Buchfink et al., 2015) mode. Carbohydrate active enzymes (CAZyme) were annotated using diamond (Buchfink et al., 2015) searches against the dbCAN database (Huang L. et al., 2018). Individual genome bins were also annotated using the KofamKOALA webserver (Aramaki et al., 2020). Average nucleotide comparisons were made with FastANI (Jain et al., 2018). Coverage information produced through MaxBin2 binning was used to assess relative abundances for genome bins and nitrogen transforming genes. Reads were mapped with Bowtie2 (Langmead and Salzberg, 2012) to obtain reads per kilobase per million mapped reads (RPKM) values that were used to conduct a chi-square test comparing the abundance of *nrfH* and *nir* (*nirK* or *nirS*) genes (Supplementary Table 5).

## Results

### 16S rRNA Gene Sequencing Reveals Shared Populations but Distinct Community Structures

The diversity and structure of the one-stage and two-stage reactor microbial community was first investigated through 16S rRNA gene sequencing. In total, 476 zero-radius operational taxonomic units (OTUs) were identified across all five samples. Much of this diversity was shared between biomass fractions (Supplementary Figure 1); for example, the one-stage and two-stage granule fractions shared 174 OTUs, accounting for 60 and 65% of the reads in each of the two libraries, respectively. Only a limited number of OTUs were unique to any one of the five biomass samples (Supplementary Figure 1) and these unique OTUs only accounted for a small proportion of the total reads in each library (Table 1). Only 54 OTUs had greater than 1% relative abundance in at least one of the biomass samples, and the detection of these 54 OTUs varied between samples, ranging from 11 in the two-stage granules (2S-granules) to 22 in the one-stage suspended (1S-suspended) library (Table 1).

**TABLE 1:** OTU counts per sample.

Sample	No. of OTUs per sample	No. of unique OTUs (% of total reads)	No. of OTUs with > 1% rel. abun. in sample (% of total reads)	Most abundant OTU (% of total reads
1S-Granules	289	12 (1.0%)	17 (74%)	OTU 2, *Brocadiales* (18%)
1S-Flocs	271	0 (0.00%)	16 (81%)	OTU 3 *Anaerolineales* (18%)
1S-Suspended	268	15 (1.2%)	22 (80%)	OTU 5 *Bacteroidetes* (11%)
2S-Granules	268	10 (0.70%)	11 (82%)	OTU 1 *Ignavibacteriales* (36%)
2S-Suspended	252	24 (3.2%)	15 (71%)	OTU 8 *Alteromonadales* (15%)

*A total of 54 OTUs were identified with greater than 1% relative abundance in one or more sample.*

**FIGURE 1:**
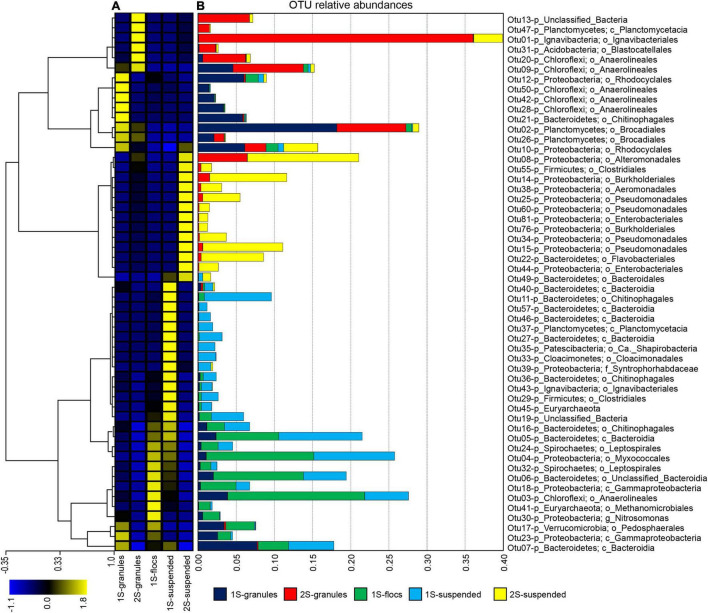
The top 54 OTUs, those with greater than 1% relative abundance in at least one biomass sample (granules, flocs, or suspended) from the one-stage (1S) and two-stage (2S) reactors. **(A)** Hierarchical clustering of OTUs (each row of the heat map) based on z-score transformation of each row. The color scale indicates z-scores from the minimum (–1.1) to the maximum (1.8) value. **(B)** The relative abundances for the top 54 OTUs in all five sequence libraries in each of the samples are indicated by stacked bars that are colored according to the biomass sample.

In total, 54 OTUs were identified that had greater than 1% relative abundance in any of the sequence libraries (Figure 1). Hierarchical clustering of z-score transformed relative abundances for these 54 most abundant OTUs (Figure 1A) separated individual taxa into five main clusters. Relative abundances of OTUs within each cluster (Figure 1B) indicated strong preferences for reactor configuration and biomass fraction. OTUs that were most abundant in the granule biomass from the one-stage and two-stage configurations formed two distinct clusters. Only four OTUs were abundant in both granule samples. OTU_09 assigned to phylum *Chloroflexi* and order *Anaerolineales* represented ∼5 and 9% of the one and two-stage granules. OTU_10 assigned to phylum *Proteobacteria* and order *Rhodocyclales* represented ∼6 and 3% of the one and two-stage granules. Anammox bacteria were primarily represented by two OTUs assigned to order *Brocadiales* that was shared between libraries. OTU_02 was more abundant, representing 18% of the sequences from the one-stage granules and only 9% from the two-stage granules. Another major difference between the granule biomass from the two reactor configurations was the relative abundance OTU_01 assigned to order *Ignavibacteriales*, which represented only 0.006% of the one-stage granule library but was the most abundant OTU in the two-stage granules with a relative abundance of 36%. Two clusters (Figure 1) were primarily composed of OTUs that tended to have high relative abundance in the suspended fraction of either the one-stage or two-stage configurations. The fifth cluster was composed of OTUs that were generally most abundant in the one-stage floc biomass sample, but these taxa tended to also be abundant within the granule and suspended fractions.

### Abundant Heterotrophic Populations Differ Between Reactor Configuration Granules

**FIGURE 2:**
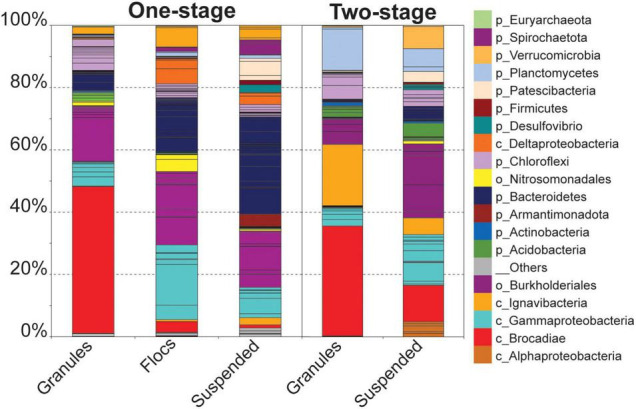
Relative coverage of genome bins identified in the three biomass fractions from the one-stage reactor sample and in the two biomass fractions from the two-stage reactor sample. Individual genome bins are indicated by stacked bars. Bins assigned to the same taxonomy are grouped and colored accordingly. Taxonomy of individual bins is represented at the phyla or higher level when appropriate (e.g., separate taxonomy for *Brocadiae* vs. *Planctomyces* for non-anammox bins.

Hybrid assembly of Illumina short-reads and Oxford Nanopore MinION long-reads resulted in improved overall metagenome assembly and binning of assembled scaffolds into genome bins than assembly using Illumina short reads alone (Supplementary Table 1). In total, assemblies generated 78 genome bins for the one-stage and 54 genome bins for two-stage configurations. Comparing the relative abundance of the bins across all five samples revealed that although many of the same type of taxa were present in all five samples, there were distinct community structures between each reactor configuration and among the biomass fractions within them (Figure 2), similar to the trend observed for OTUs (Figure 1).

**FIGURE 3:**
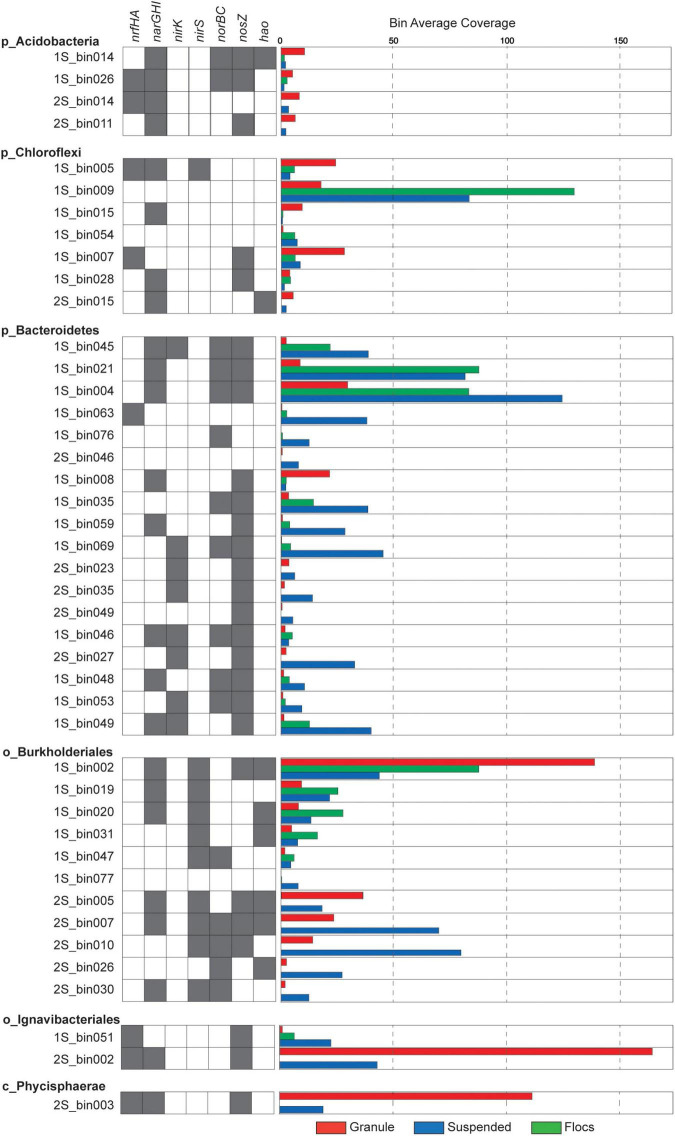
Presence of nitrogen transforming genes is indicated by gray shaded boxes and relative coverage for individual MAGs in the different samples is indicated by the colored bars.

Unlike the granular biomass within the one-stage system which was dominated by two bins assigned to *Brocadiae* and *Burkholderiales*, the two-stage granules had three dominant bins assigned to *Brocadiae*, *Ignavibacteria*, and *Planctomyces* (class *Phycisphaerae*). As in the 16S rRNA gene sequence data, the abundance of *Ignavibacteria* was a distinguishing characteristic between the two reactor configurations. Coverages for bins assigned to *Bacteroidetes* were substantially greater in the one-stage samples, with relative abundances increasing from granules to flocs, to suspended fractions. *Burkholderiales* and *Chloroflexi* bins were also abundant in all five samples. Forty-three of the recovered high-quality MAGs (>80% completeness and < 20% contamination), (Supplementary Tables 2, 3) belonging to the six most abundant lineages of heterotrophs were selected for further analysis (Figure 3). In general, *nar* and *nos* genes were distributed among the MAGs from all six lineages, whereas *nrf*, *nir, and nor* genes had more limited distributions.

### Abundant Ignavibacteria and Phycisphaerae Perform Dissimilatory Nitrate Reduction to Ammonia in the Two-Stage Granules

Of these 43 MAGs, 2S_bin002 (assigned to *Ignavibacteria*) and 2S_bin003 (assigned to Phycisphaerae) were the most abundant, non-Anammox MAGs recovered from the two-stage system (Figure 3). The *Ignavibacteria* MAG contained *narGHI* and *nrfAH* genes, plus *nosZ*. The presence of genes annotated to CAZyme classes for cellulose binding modules (CBM), carbohydrate esterases (CE), glycoside hydrolases (GH), glycosyltransferases (GT), and polysaccharide lyases (PL) suggest the use of carbohydrates by this *Ignavibacteria* population. Also, the presence of ABC transporter genes for several oligosaccharides (e.g., maltose, galactose, mannitol, maltose, and cellobiose) seems to confirm this. However, this MAG was missing the isocitrate dehydrogenase gene, potentially indicating an incomplete citric acid cycle. The presence of alcohol dehydrogenase suggests the capacity for fermentation. Indeed, two component regulators for citrate fermentation (*citB* family) were also present. The *Ignavibacteria* MAG also contained genes for a cytochrome *bd* complex, which function as quinol or menaquinol oxidases, and may be beneficial for combating oxidative and nitrosative stress (overproduction of nitric oxide) by anaerobic bacteria or it may also serve as a high affinity terminal oxygen reductase (Borisov et al., 2011; Giuffrè et al., 2014; Leclerc et al., 2015). Together, these findings suggest that this *Ignavibacteria* species is well adapted to the anoxic conditions within the second stage of the two-stage reactor granular biomass but not in the aerated one-stage system.

The second most abundant of these two MAGs was 2S_bin003, assigned to phylum *Planctomycetes* and class *Phycisphaerae*. The *narH* and *nrfAH* genes were identified in this *Phycisphaerae* MAG, potentially equipping this organism with the capacity for DNRA, and the *nosZ* gene was also found. Genes were assigned to CAZyme classes CBM, CE, GH, GT, and PL suggesting the importance of Carbohydrate metabolism. The 2S_bin003 MAG also had genes for a complete citric acid cycle, oxidative pentose phosphate pathway, and the Embden-Meyerhof pathway. ABC transporters for ribose, galactofuranose, oligopeptides, plus lipoproteins and lipopolysaccharides, indicate that these are likely important carbon sources. The presence of a *cbb*_3_-type cytochrome *c* oxidase, superoxide dismutase, and the oxygen sensing two component regulator *fixL* suggest the capacity for aerobic metabolism under microoxic conditions. Additionally, 2S_bin003 has two component regulatory genes related to regulation of biofilm formation (e.g., EPS biosynthesis and flagella).

### Burkholderiales Are the Dominant Denitrifiers in the One-Stage System

**FIGURE 4:**
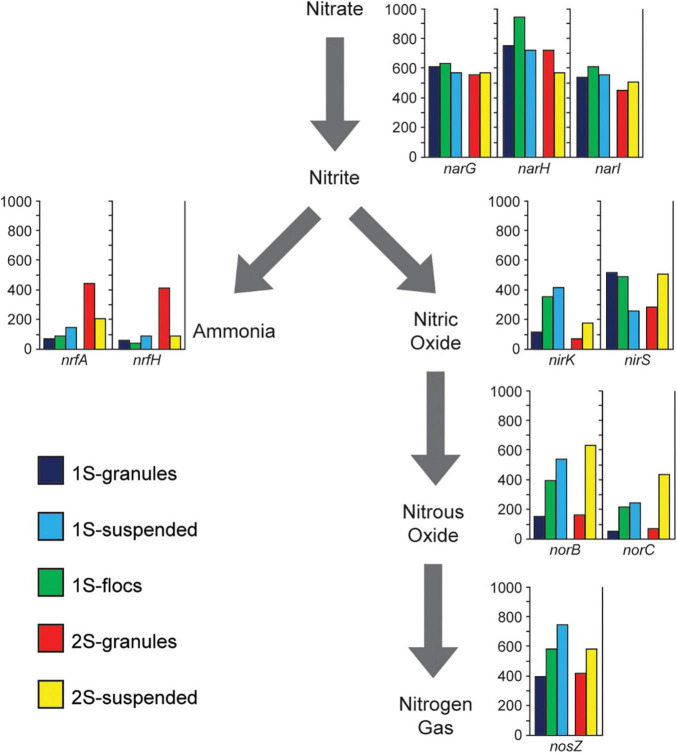
Presents metagenome coverage (*y*-axis of graphs) for Nitrogen transforming genes (*nrfA, nrfH, narG, narH, narI, nirK, nirS, norB, norC, and nosZ*) assigned to heterotrophic populations for the five sequence libraries representing the granules, flocs, and suspended biomass from the one-stage system (1S) and the granules and suspended biomass from the two-stage system (2S) excluding genes associated with the Anammox bacteria.

The 11 MAGs assigned to phylum *Proteobacteria* and order *Burkholderiales* exhibited varying abundance patterns across the biomass fractions (Figure 4). None of them contained the *nrfAH* genes needed for DNRA, while seven of these MAGs possessed at least one of the *narGHI* genes and a *nirS* gene but varied in their potential to reduce NO or N_2_O. All of the *Burkholderiales* MAGs that contained a *nosZ* gene also had a *nosR* gene indicating that they belong to clade I, indicative of their potential role as canonical denitrifiers (Hein and Simon, 2019). Average nucleotide identity (ANI) comparisons among *Burkholderiales* MAGs identified two similar populations shared among reactor configurations. The *Burkholderiales* MAG (2S_bin005), and another bin (1S_bin016) that was just below the 80% completeness threshold, were both more abundant in the granule libraries and had a 97.8121% ANI. One of the most abundant two-stage MAGs, the suspended biomass enriched 2S_bin007, which was the only MAG with a complete denitrification pathway, had 99.0548% ANI with the granule enriched one-stage MAG, 1S_bin002, which was also the most abundant non-Anammox granule enriched MAG recovered from the one-stage reactor biomass. These two MAGs, which likely represent the same species, possessed both *napAB* and *narGHI* nitrate reductases, as well as the *nirS* and *nosZ* genes, but only the 2S_bin007 had the *norB* gene, which could be due to incomplete assembly and binning of 1S_bin002. Having both *narGHI* and *napAB* genes may enable it to perform denitrification across a range of nitrate and oxygen concentrations (Papaspyrou et al., 2014; Ji et al., 2015). The presence of the *hao* gene in this and other recovered Burkholderiales MAGs may also provide capacity to oxidize hydroxylamine, as has been reported for other heterotrophic denitrifiers (Otte et al., 1999; Chen and Ni, 2011).

Because the MAG 1S_bin002 affiliated with the *Burkholderiales* was such an abundant member of the one-stage reactor microbial community and represented a major component of the genomic potential for denitrification in that reactor configuration, we looked further into potential genomic features that would play a role in its success within the one-stage PNA system. In addition to the *cbb*_3_-type cytochrome *c* oxidase, both 1S_bin002 and 2S_bin007 had the proton translocating cytochrome *bc*_1_ complex necessary for respiratory denitrification (Trumpower, 1990). Both MAGs contained *sox* and *dsr* genes which would confer the ability to perform lithotrophic denitrification using reduced sulfur compounds (Beller et al., 2006; Shao et al., 2010) which are produced through sulfate reduction and organic matter degradation (Lin et al., 2018). Potential carbon sources utilized by 1S_bin002 and 2S_bin007 were indicated by the presence of ABC transporter genes for phospholipids, lipopolysaccharides, lipo-oligosaccharides, branched-chain amino acids, and glutamate. Both MAGs possessed complete pathways for glycolysis, the citric acid cycle, fatty acid beta-oxidation, and benzoyl-CoA degradation. Additionally, the presence of nitrogen sensing two-component regulatory genes *narXL* and *ntrY* along with oxygen sensing *fixLJ* and redox sensing *regAB* would allow this organism to sense and adjust to changes in nitrate and oxygen availability. Overall, the organism represented by the two MAGs, 1S_bin002 and 2S_bin007, is likely a versatile heterotroph with the ability to adapt to oxic, microoxic, and anoxic conditions.

### Acidobacteria, Bacteroidetes, and Chloroflexi Had Varying Capacity for N Transformation

All four MAGs assigned to the phylum *Acidobacteria* were most abundant in the granule fraction of their respective reactor source, and all contained at least one copy of each of the *narGHI* set of genes, but none contained *nirS* or *nirK* genes. Both the 1S_bin026 and 2S_bin014 contained *nrfGH* genes, indicating their potential role in DNRA. Two of the *Acidobacteria* MAGs contained *norB* and three had *nosZ*. Three of these MAGs were of the order *Bryobacteriales* (1S_bin014, 1S_bin026, and 2S_bin014) but all pairwise comparisons of average nucleotide identity (ANI) (Jain et al., 2018) were between 77 and 79%. The remaining *Acidobacteria* MAG was assigned to the order *Pyrinomonadales* (2S_bin011). Carbohydrate active enzyme (CAZyme) annotations showed that among all the recovered MAGs, the three *Bryobacteriales* MAGs had the highest numbers of enzymes assigned to GH, CE, and GT classes. In addition, two had the highest number of PL among all recovered MAGs. All four *Acidobacteria* MAGs also contained proteins with CBM, and copper-dependent lytic polysaccharide monooxygenases (LPMOs). Together, these annotations suggest that Anammox reactor granule associated *Acidobacteria* are primarily involved in the degradation of polysaccharides and may utilize nitrate to oxidize sugars under oxygen limitation but vary in their capacity to further reduce nitrite. This is consistent with genomic analyses of terrestrial *Acidobacteria*, that have the capacity to utilize carbohydrates across oxygen gradients (Eichorst et al., 2018).

Eighteen of the recovered MAGs were assigned to the *Bacteroidetes* phylum and they also varied in their potential for nitrogen transformation. All but two of these MAGs were most abundant in the suspended biomass fraction from either reactor configuration. 1S_bin008 was more abundant in the granule biomass and contained both *narGHI* and *nosZ* genes. 1S_bin046 was abundant in the floc biomass and potentially had the capacity for complete denitrification. The 1S_bin004 had the highest assembly coverage of all MAGs in the one-stage suspended metagenome, but the *nir* gene was not detected, which could be a result of either an incomplete assembly or an incomplete denitrification pathway. None of the three *Bacteroidetes* MAGs recovered from the two-stage reactor had *narGHI* genes, but two had the *nirK* and *nosZ* genes. Only one *Bacteroidetes* MAG (1S_bin063) had *nrfAH* genes, however, it had none of the other nitrogen transformation genes. Overall, the recovered *Bacteroidetes* MAGs also had abundant CAZymes, which together with a general greater abundance in the one-stage suspended fraction suggests their primary function as heterotrophs with some of them having the potential for nitrogen reduction.

All but one of the seven MAGs assigned to the phylum *Chloroflexi* were recovered from the one-stage reactor biomass, and these MAGs varied in their abundance across the different biomass fractions. The *Chloroflexi* MAG, 1S_bin009, had the highest read coverage in the floc biomass but did not have any annotated nitrogen respiratory genes. The 1S_bin005 MAG was most abundant in the granule biomass and possessed both *narGHI* and *nrfAH* genes, suggesting a potential role in DNRA. 1S_bin007, had the *nrfAH* genes, but was missing the *narGHI* genes. In general, the *Chloroflexi* MAGs had varying capability to reduce nitrate (four of the seven had *narGHI* genes), and only one MAG possessed a *nirS* gene, suggesting that some of these populations may play a role in the nitrite loop, reducing nitrate produced by Anammox, but not in complete denitrification. Filamentous *Chloroflexi* are commonly identified in WWTS where they function as sugar fermenters and may also play an important role in biofilm formation (Nierychlo et al., 2019; Speirs et al., 2019).

### N-Transformation Gene Abundance Differs Between Reactor Configurations

In order to assess the overall potential functional differences in nitrogen transformation between the different biomass fractions in the two PNA reactor configurations, different genes involved in denitrification and DNRA were identified within the assemblies. Genes assigned to the two *Brocadiales* MAGs were excluded to focus the analysis on the potential role of heterotrophic populations. The relative abundance (based on coverage data) for genes in the different sequence libraries corresponding to the different biomass fractions is shown in Figure 4. *NarGHI* genes were the most abundant nitrogen transforming genes across all sequence libraries from both reactor configurations, however, the abundances of genes for DNRA and denitrification among heterotrophic populations differed between reactor configurations. Comparing RPKM values for binned scaffolds with either a *nrfH* or a *nir* (*nirK* or *nirS*) gene (Supplementary Table 5) indicated a significant difference between granules from the one-stage and two-stage PNA configurations (*X*^2^ = 79.72, *p* = 4.3E-19). The relative coverages of *nrfA* and *nrfH* were much higher in the two-stage granule metagenome than in any of the other samples, suggesting a greater potential for DNRA activity by heterotrophs within that reactor configuration than in the one-stage system. This is primarily due to the abundance of the *Ignavibacteria* MAG (2S_bin002) and *Phycisphaerae* MAG (2S_bin003) (Figure 3). The relative abundance of *nirS* genes showed opposing trends, decreasing in abundance from granule, to floc, and to suspended biomass in the one-stage configuration, but were more abundant in the two-stage suspended biomass than in the two-stage granules. Both *norBC* and *nosZ* genes had lower relative abundance within the granule fraction than within either of the suspended fractions or the floc fraction in the one-stage system.

## Discussion

In this study, we utilized 16S rRNA gene sequencing and metagenomic analysis to compare the microbial communities of two distinct PNA reactor configurations, a one-stage and a two-stage configuration. We focused the analysis of recovered MAGs on the genomic properties of high abundance populations that differed in abundance between reactor configurations to develop hypotheses of how the selective pressures imposed by the distinct reactor configurations may impact the microbial community structures and their potential functions. Previous studies of PNA bioreactors have observed microbial community structures over time, with some stability observed in bench scale reactors (Chen et al., 2017) and conversely, community dynamics have been observed that correlated with changes in influent ammonia and nitrite concentrations (Huang Q. et al., 2018). The community structure and potential function of PNA granules is also impacted by granule size, with nitrifiers being more abundant in smaller granules and larger granules harboring greater species diversity (Luo et al., 2017). Together, these studies indicate that substrate composition and biofilm structure impose selective pressures that impact the heterotrophic microbial communities within PNA reactors. The reactors sampled in this study have been maintained under long-term stable operating conditions (van Dongen et al., 2001; Abma et al., 2007; van der Star et al., 2007; Klein et al., 2018), suggesting that a stable microbial community has emerged under the unique environmental conditions imposed by each system.

The two reactor configurations examined in this study harbored a single, nearly identical Anammox MAG representing the same species, however, the accessory heterotrophic populations, represented by the OTUs and recovered MAGs, exhibited different abundance patterns among all the samples (Figures 1–3), suggesting that the one and two-stage reactor configurations impose different selective pressures that provide distinct niches among the different biomass fractions (granules, flocs, and suspended cells). A consequence of these selective pressures is the observed differences in the abundances for key nitrogen respiratory genes among heterotrophic populations (Figure 3), favoring denitrification within the one-stage PNA reactor and DNRA in the two-stage reactor. There were some discrepancies observed between the amplicon and metagenomic analyses; lower relative abundances were observed for OTUs assigned to Anammox bacteria in all samples when compared to their relative abundances in the metagenome libraries. Alternatively, the relative abundance of an *Ignavibacteria* OTU and MAG exhibited the opposite trend. This can likely be attributed to the effects of PCR primer biases in PNA reactor communities (Orschler et al., 2019) as extraction biases would similarly affect amplicon and shotgun metagenome sequencing. Nevertheless, similar taxa were identified in the amplicon and sequence libraries for the different biomass fractions in both reactor configurations.

### Reactor Configuration Determines Niche Availability

The primary environmental parameter differentiating the one-stage and two-stage PNA reactor configurations is the presence of oxygen. The one-stage PNA reactor configuration utilizes continuous aeration to promote partial nitrification, which produces nitrite for Anammox bacteria. The activities of both nitrifiers and aerobic heterotrophs serve to reduce oxygen penetration into the one-stage granules, therefore supporting an anoxic niche space while under constant aeration. The distribution of nitrifying populations observed in this study is in alignment with prior observations of one-stage PNA systems, in which nitrifier growth was localized to the granule periphery where oxygen was available (Winkler et al., 2012b). The most abundant *Nitrosomonas* MAG and OTU had its highest relative abundance in the one-stage flocs library, with lower relative abundance in the granules, implying limited aerobic niche space within the biofilm of the granules. The anoxic environment imposed by the second stage of the two-stage PNA system resulted in limited detection of nitrifiers. A *Nitrosomonas* MAG identified in this study was only detected at low levels in the two-stage suspended fraction. Since the second stage of the two-stage PNA bioreactor is operated under anoxic conditions, it is likely that this biomass transferred from the first, oxic, stage during reactor operation, but was unlikely to be active, and is consistent with previous reports of biomass transferring between two-stage PNA bioreactor stages (van Dongen et al., 2001). Nitrite oxidizers were not identified in either reactor configuration, indicating that operational strategies for both of the PNA systems successfully mitigated their growth.

The one-stage reactor conditions would likely promote the growth of aerobic heterotrophs (some of which may be facultative anaerobes) within flocculent biomass or as suspended cells where diffusion of oxygen would be less limiting than in the biofilm structure of granules. Indeed, the relative abundances for all but one of the *Bacteroidetes* MAGs recovered from the one-stage system was lowest in the granules and highest in the suspended fractions. Reactor operational strategies that impact granule size will also impact the resulting community structures, as larger granules will provide relatively more anoxic volume than smaller granules (Nguyen Quoc et al., 2021). Additionally, microbial growth within granules occurs as colonies (Gonzalez-Gil et al., 2015), rather than homogeneous layers, which would provide some opportunity for a more heterogeneous redox environment within one-stage PNA granules than within the uniformly anoxic environment of granules in the second stage of a two-stage PNA reactor configuration. Thus, the anoxic (second) stage of a two-stage PNA reactor likely presents a more stable redox environment that would select for anaerobic metabolisms within the granules, whereas the suspended biomass obtained in this reactor would likely contain a mixture of bacteria transferred along with the wastewater from the first stage as well as native bacteria that shed from granule surfaces.

The observed differences in community structure between granules from the two PNA configurations seems to support this interpretation. Besides the Anammox bacteria, there were a greater number of low abundance taxa present in the one-stage PNA granules than in the two-stage granules, which were dominated by two heterotrophic taxa. This suggests that there is a greater diversity of available niches within the one-stage granules, whereas the anoxic environment of the two-stage PNA system may provide a stable anaerobic niche space. The diversity of heterotrophic populations within the one-stage PNA system may also relate to the availability of oxygen for aerobic respiration of carbon sources. This factor may have two main impacts on the structure of PNA reactor microbial communities (Supplementary Figure 2). Within the one-stage PNA system, organic carbon substrates in the influent waste stream would likely be rapidly consumed through aerobic metabolism; this is supported by the observation of higher relative abundances for heterotrophic taxa within the floc and suspended fractions than in the granules and prior reports of the rapid growth and migration of heterotrophs out of granular biofilms into the bulk liquid (Weissbrodt et al., 2013). This rapid depletion and the associated concentration gradients would reduce the overall availability of externally supplied organic carbon sources for heterotrophic denitrification and DNRA activities by microorganisms growing in the granules within the one-stage PNA system. Additionally, heterotrophic populations within the one-stage system would preferentially utilize oxygen over nitrate or nitrite as the terminal electron acceptor as it yields more energy for cell growth. Together, these activities would reduce the carbon to nitrate ratio and add selective pressure for denitrification within a one-stage PNA configuration.

Furthermore, the utilization of organic carbon within anoxic zones of the one-stage PNA granules may be more complex than the competition between aerobic and anaerobic heterotrophs, as Anammox also consume organic carbon for DNRA (Castro-Barros et al., 2017). Carbon limited conditions within one-stage PNA granules may favor DNRA by Anammox over heterotrophic populations (Güven et al., 2005; Winkler et al., 2012a,b). Anammox bacteria had higher relative abundance in the one-stage granules than in the two-stage granules, which may not only result from the competition for available nitrate, nitrite, and organic carbon between aerobic and anaerobic heterotrophs performing denitrification or DNRA, but also between Anammox and heterotrophs. One caveat to the interpretation of relative abundances for populations and their functional genes is that it may not reflect the true relative activity of their respective processes. Relative abundances may reflect their respective biomasses, however, the yield from carbon oxidation may result in relative abundance measures for heterotrophic populations, as opposed to autotrophic populations Anammox and nitrifiers, that overshadow their contribution to net reactor processes.

### Niche Adaptations Differentiate Denitrifying and Dissimilatory Nitrate Reduction to Ammonia Organisms

The ability of microorganisms to exploit multiple niches along redox gradients was exemplified by a single species assigned to order *Burkholderiales* and family *Rhodocyclaceae* and represented by two highly similar MAGs (1S_bin002 MAG and 2S_bin007, also OTU10). This organism was more abundant in the suspended fraction than in the granules in the two-stage system but was the second most abundant population in the one-stage granules after Anammox. Its genomic content suggests an ability to fine tune its physiology to varying redox conditions, such as by using different terminal oxidases or switching between *nap* and *nar* nitrate reductases depending upon oxygen and nitrate concentrations. Dynamic environmental conditions may play an important role in the competition between heterotrophic denitrifying facultative anaerobes, and organisms capable of performing fermentation and respiratory DNRA. The ability to alter its affinity to nitrate may indeed allow for successful competition with other denitrifiers and with organisms performing DNRA under conditions of different carbon and nitrate concentrations and carbon to nitrate ratios (Jia et al., 2020). In contrast, the *Ignavibacteria* MAG, which was highly abundant in the two-stage granules but not in the one-stage granules, may potentially be limited to anaerobic niches that favor a fermentative lifestyle, and may be better able to compete with Anammox bacteria for carbon and nitrate or nitrite when not having to compete with facultative anaerobic heterotrophs. *Ignavibacteria* have been associated with DNRA in estuary sediments (Bu et al., 2017) in addition to commonly being identified in Anammox reactors (Guo et al., 2016; Speth et al., 2016; Lawson et al., 2017; Wang et al., 2019). A single sequenced isolate, *Ignavibacterium album*, was thought to be a strict fermentative anaerobe, but genomic evidence suggests greater metabolic versatility (Iino et al., 2010; Liu et al., 2012). Yet, knowledge of the diversity within this clade is limited, and the extent to which the single isolate obtained from a terrestrial hot spring is representative of *Ignavibacteria* found in WWTS remains to be determined. In addition to carbon and nitrate availability, the transient presence of oxygen may favor denitrification over DNRA, as has been reported for microbiota inhabiting the root zones of plants in wetland sediments (Matheson et al., 2002).

Overall, the outcome of competition between heterotrophs performing DNRA and denitrification can have deleterious impacts on PNA reactor performance in terms of reduced nitrogen removal or increased aeration requirements owing to production of ammonia as well as the potential effects of competition for nitrate or nitrite between DNRA performing heterotrophs and Anammox bacteria (Keren et al., 2020). Resource-ratio modeling indicated that kinetic factors, carbon to nitrate ratio, and total carbon and nitrate loading can impact the competitive outcome, yet within these models there are also conditions in which coexistence can occur (Jia et al., 2020). Furthermore, experiments indicate that the type of carbon source can also affect the competition (van den Berg et al., 2016, 2017; Carlson et al., 2020). Taken together, these finding indicate that competition between DNRA and denitrification is highly nuanced. Indeed, the redox gradients within Anammox granules likely contain diverse microenvironments that in turn support diverse DNRA or denitrifying organisms. Furthermore, understanding the individual life strategies of these organisms provides valuable clues as to why specific taxa may or may not be successful within PNA systems and can lead to operational strategies that select for more desirable function.

## Conclusion

In conclusion, heterotrophic populations have been consistently identified in different online and lab scale PNA systems, where they may provide important physical, chemical, or biological functions. For example, heterotrophic denitrifying populations may perform beneficial ecological services within PNA systems, either by converting nitrate produced by Anammox back to nitrite, or by reducing nitrate to dinitrogen gas. DNRA performing heterotrophs, however, may represent an undesirable function within these communities, reducing nitrate or nitrite back to ammonia, thereby reducing the overall systems nitrogen removal performance and increasing the aeration demands, and potentially competing with Anammox bacteria for substrates and space. The one-stage and two-stage PNA granules examined in this study differed markedly in terms of the abundance of heterotrophic taxa associated with denitrification and DNRA, which may be conventionally attributed to the competition for carbon and nitrate under oxic and anoxic conditions. While this study identifies specific taxa and the genomic features that may promote success within the different environments imposed by the distinct PNA reactor configurations, further work is needed to examine the competitive interactions among autotrophic or organotrophic Anammox and heterotrophic populations. A broader examination of available metagenomes derived multiple reactor configurations may provide more insight into the selective pressures that impact the competition between denitrification and DNRA metabolisms. Reported analysis of bench scale reactors have shown that in some instances DNRA may become more prevalent and negatively impact nitrogen removal performance (Keren et al., 2020), while in other reactors the abundance of *nrf* genes remains lower (Wang et al., 2019). The type of carbon source has also been implicated in determining the competitive outcome of denitrification and DNRA (van den Berg et al., 2016, 2017), so further comparisons of Anammox reactor metagenomes should assess the impacts of both municipal and agricultural waste streams that may supply distinct amounts and types of organic substrates. Furthermore, the role of cross-feeding within Anammox granules has been suggested by metagenomic analysis (Zhao et al., 2018), but empirically testing and quantifying this activity may identify interactions between Anammox and heterotrophic populations, and whether those interactions are influenced by reactor operational strategies. Finally, granule size impacts the ratio of oxic and anoxic niche spaces (Nguyen Quoc et al., 2021) within aerated reactors, so an investigation into the effects of granule size on the abundance of denitrifying and DNRA performing heterotrophs within one-stage PNA reactors may provide further insight into the selective pressures determining competitive outcomes as well as inform reactor operational strategies to favor improved nitrogen removal performance.

## Data Availability Statement

The datasets presented in this study can be found in online repositories. The names of the repository/repositories and accession number(s) can be found below: https://www.ncbi.nlm.nih.gov/, PRJNA597196.

## Author Contributions

SB, KH, DS, and M-KW contributed to research design and manuscript preparation. SB performed all laboratory and bioinformatics analyses. All authors contributed to the article and approved the submitted version.

## Conflict of Interest

The authors declare that the research was conducted in the absence of any commercial or financial relationships that could be construed as a potential conflict of interest.

## Publisher’s Note

All claims expressed in this article are solely those of the authors and do not necessarily represent those of their affiliated organizations, or those of the publisher, the editors and the reviewers. Any product that may be evaluated in this article, or claim that may be made by its manufacturer, is not guaranteed or endorsed by the publisher.
