# Supplementary material for: Metagenomic Insights Into Competition Between Denitrification and Dissimilatory Nitrate Reduction to Ammonia Within One-Stage and Two-Stage Partial-Nitritation Anammox Bioreactor Configurations
Source: Front Microbiol. 2022 Apr 25;13:825104. doi: 10.3389/fmicb.2022.825104 (PMC9083452; doi:10.3389/fmicb.2022.825104)
Supplement: Supplementary file 1 [file Data_Sheet_1.PDF]

Supplementary Material

1 Supplementary Figures and Tables

1.1 Supplementary Figures

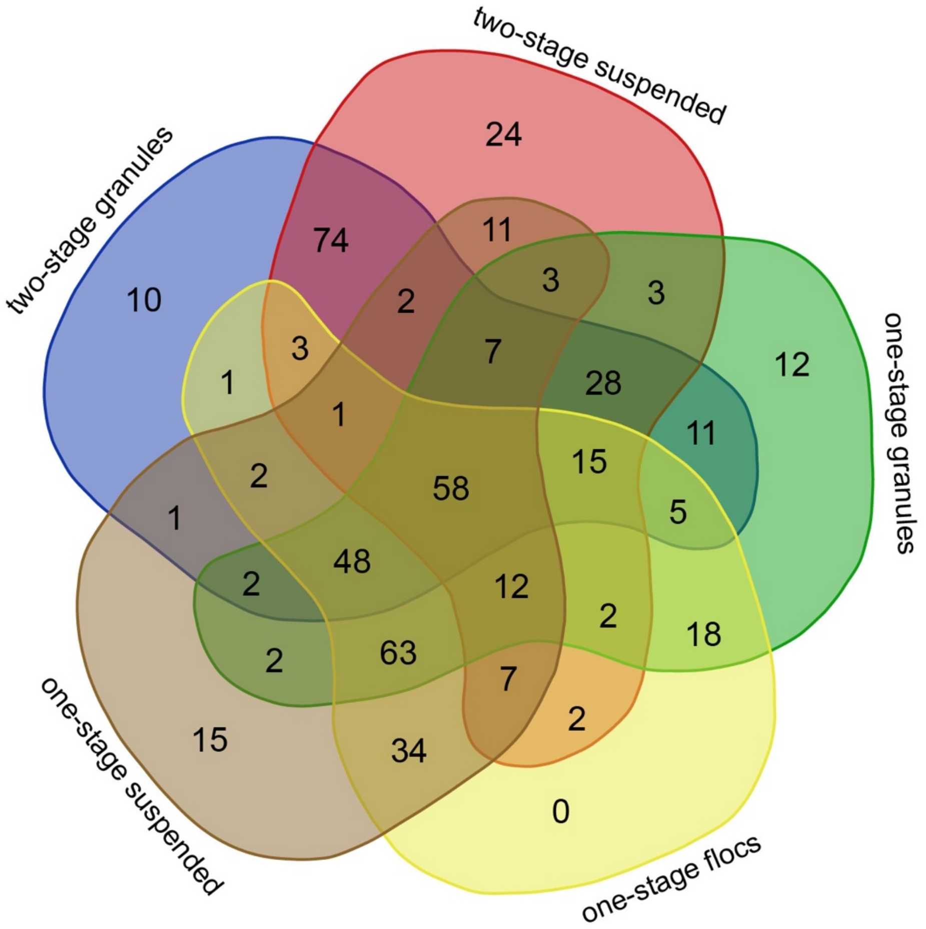

Supplementary Figure S1. Venn diagram depicting the detection of 476 zero-radius operational taxonomic units (OTUs) across the five sequence libraries obtained from granules, flocs, and suspended biomass sampled from the one-stage partial nitritation Anammox (PNA) bioreactor and the granules and suspended biomass sampled from the two-stage PNA bioreactor.

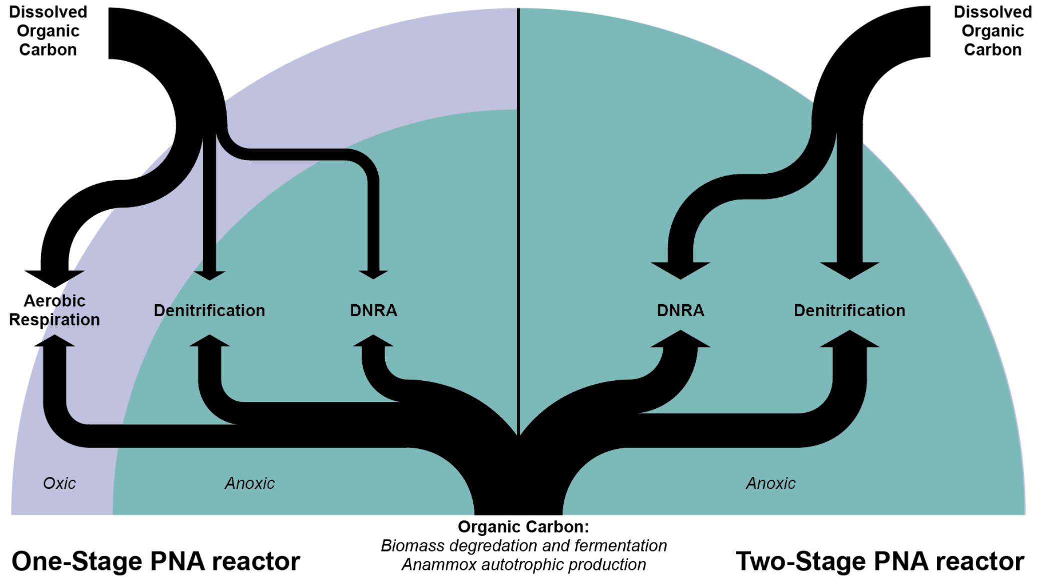

Supplementary Figure S2. Graphical representation of potential carbon utilization by aerobic and anaerobic metabolisms within the oxic and anoxic zones of one-stage PNA reactor granules and the uniformly anoxic environment of two-stage PNA reactor granules. Carbon sources include the potential products of biomass degradation within the granules and diffusion of dissolved organic carbon into the granules. The one-stage PNA granules provide a larger space for heterotrophic populations that can exploit the oxygen gradient by utilizing both oxygen and nitrate or nitrite as terminal electron acceptors. Whereas the two-stage PNA granules provide a homogenous anoxic environment that selects for anaerobic metabolisms. The arrow width is for illustrative purposes and not based on experimental data.

1.2 Supplementary Tables

Table S1. Metagenome Assembly and Analysis for Two Anammox Reactor Configurations

**Illumina Sequencing Libraries**	**One-stage**	**Two-stage**
Granules Library (reads)	31,704,368	37,315,146
Flocs Library (reads)	25,246,956	---
Suspended Library (reads)	29,807,818	36,595,460
Combined Sequence Libraries (reads)	86,759,142	73,910,606
Quality Filtered Sequence Libraries (reads)	78,212,954	68,101,916
**MinION Sequencing Libraries**	**One-stage**	**Two-stage**
Granules Library (reads/bases)	542,466 / 1,749,795,291	355,793 / 1,322,045,671
Flocs Library (reads/bases)	140,631 / 333,861,736	---
Suspended Library (reads/bases)	161,818 / 375,013,591	308,050 / 1,105,285,823
Combined and corrected (reads/bases)	8,604 / 142,067,376	10,579 / 139,793,554
Trimmed (reads/bases)	7,070 / 97,239,560	8,484 / 89,782,431
Assembled (contigs/bases/N50)	14 / 3,595,813	58 / 7,642,686 / 613,783
**metaSPADES assembly (Illumina)**	**One-stage**	**Two-stage**
Total Assembly Length	251,042,245 bp	199,704,920 bp
# Scaffolds	33,488	30,430
Assembly N50	14,846 bp	10,450 bp
Longest Scaffold	1,829,904 bp	482,456 bp
**hybridSPADES assembly (Illumina + MinION)**	**One-stage**	**Two-stage**
Total Assembly Length	288,375,887 bp	243,267,605 bp
# Scaffolds	33,730	31,366
Assembly N50	22,878 bp	15,673 bp
Longest Scaffold	2,460,838 bp	2,954,805 bp
**metaSPADES assembly (Illumina Read Mapping)**	**One-stage**	**Two-stage**
One-stage Granules	70.63%	47.36%
One-stage Flocs	78.20%	12.58%
One-stage Suspended	74.49%	7.83%
Two-stage Granules	33.80%	79.63%
Two-stage Suspended	14.70%	65.92%
**hybridSPADES assembly (Illumina Read Mapping)**	**One-stage**	**Two-stage**
One-stage Granules	74.24%	48.6%
One-stage Flocs	81.27%	13.42%
One-stage Suspended	77.66%	9.05%
Two-stage Granules	35.47%	82.19%
Two-stage Suspended	15.66%	70.03%
**metaSPADES assembly - MaxBin2 Binning**	**One-stage**	**Two-stage**
Bins	83	50
Binned Contigs	28,676 (85.6%)	25,564 (84.0%)
Unbinned Contigs	4,812 (14.4%)	4,866 (16.0%)
Binned Length	227,634,129 (90.7%)	175,593,697 (87.9%)
Bins ≥ 90% complete	38	21
**hybridSPADES assembly - MaxBin2 Binning**	**One-stage**	**Two-stage**
Bins	78	54
Binned Contigs	30,281 (85.8%)	25,572 (81.5%)
Unbinned Contigs	5,026 (14.2%)	5,794 (18.5%)
Binned Length	263,462,190 bp (92.7%)	210,167,126 bp (86.4%)
Bins ≥ 90% complete	39	25

Table S2. Bin Stats

BinID	Completeness	Contamination	Strain Heterogeneity	Genome Size (bp)	# Scaffolds	N50 (scaffolds)	Longest scaffold (bp)	GC%
one-stage_bin001	99.66	1.65	0	3,258,604	2	2,191,493	2,191,493	42.3
one-stage_bin002	87.57	0.93	33.33	2,958,761	121	47,019	157,219	66.8
one-stage_bin003	95.8	14.94	8.45	4,544,425	81	624,848	944,449	64.1
one-stage_bin004	100	0.48	0	2,762,551	9	2,460,838	2,460,838	39.9
one-stage_bin005	90.91	1.27	0	3,325,528	62	95,994	250,188	60.5
one-stage_bin006	98.65	1.35	0	4,496,899	16	2,426,276	2,426,276	57.8
one-stage_bin007	90.91	1.82	0	4,326,164	141	95,514	673,360	59.8
one-stage_bin008	98.01	0.51	50	3,615,894	37	219,217	717,466	41.8
one-stage_bin009	99.09	0.91	0	2,707,135	13	893,338	1,164,123	54.2
one-stage_bin010	80.53	13.56	78.26	3,837,156	430	16,787	91,959	66.2
one-stage_bin011	97.18	3.61	4	3,755,982	32	590,293	1,022,580	66.1
one-stage_bin012	94.72	54.32	1.05	5,421,459	684	17,504	253,298	69.8
one-stage_bin013	99.97	1.92	0	2,279,876	11	285,093	397,899	50.8
one-stage_bin014	96.86	1.32	0	6,973,541	228	50,149	185,148	61.9
one-stage_bin015	88.64	7.27	12.5	3,219,444	187	28,970	89,604	49.2
one-stage_bin016	79.37	12.24	64	2,653,132	350	12,647	49,228	70.4
one-stage_bin017	52.63	5.26	0	3,551,905	259	39,665	111,554	70.5
one-stage_bin018	93.1	52.28	2.04	5,414,483	761	18,662	89,194	67.4
one-stage_bin019	83.41	1.03	64.29	3,095,168	36	258,117	645,429	65.6
one-stage_bin020	83.89	3.04	0	3,090,493	110	57,187	127,610	70.2
one-stage_bin021	100	0.95	0	3,610,458	48	281,664	645,041	36.1
one-stage_bin022	64.94	0.41	0	2,180,155	356	7,521	29,611	53.3
one-stage_bin023	93.97	61.59	0	5,714,365	1,036	6,384	92,578	42.7
one-stage_bin024	59.48	5.17	0	2,812,011	536	7,110	42,779	70.6
one-stage_bin025	48.97	14.47	7.41	3,341,467	791	4,233	61,737	52.2
one-stage_bin026	97.41	17.97	4.35	5,733,001	716	12,788	69,290	58.8
one-stage_bin027	57.34	11	0	3,961,901	1,094	3,744	17,266	62.5
one-stage_bin028	93.45	11.29	12.5	4,136,162	608	9,838	94,780	64.5
one-stage_bin029	71.55	25.5	2.04	3,410,866	933	3,745	16,904	72.2
one-stage_bin030	93.75	6.82	0	4,509,350	121	121,179	287,004	63.5
one-stage_bin031	85.61	3.29	33.33	3,857,315	169	58,518	237,902	70.6
one-stage_bin032	97.2	47.61	2.15	5,495,209	178	139,679	591,939	54.4
one-stage_bin033	22.99	4.31	0	1,640,223	481	3,461	15,228	61.6
one-stage_bin034	77.91	42.19	0.83	5,800,123	1,493	3,952	67,612	66.8
one-stage_bin035	96.95	2.97	33.33	3,724,515	74	91,825	216,018	37.4
one-stage_bin036	92.1	3.87	0	6,136,092	25	509,931	1,142,674	54.5
one-stage_bin037	96.48	0.37	0	2,391,878	24	279,704	444,880	49.1
one-stage_bin038	98.49	0.24	0	2,334,805	75	66,068	150,748	48.7
one-stage_bin039	20.83	12.5	0	4,736,908	1,416	3,226	49,572	70.9
one-stage_bin040	75.47	36	3.51	4,929,083	1,240	4,370	23,112	64.4
one-stage_bin041	66.77	35.28	0	3,283,290	1,006	3,186	27,186	62.2
one-stage_bin042	97.44	2.56	33.33	3,779,422	84	200,025	966,920	68.9
one-stage_bin043	89.39	4.03	75	4,637,153	98	159,260	544,466	71.8
one-stage_bin044	83.25	20.15	2	5,583,777	987	7,192	45,920	72.1
one-stage_bin045	98.91	3.28	16.67	3,480,968	66	100,895	463,417	38.8
one-stage_bin046	94.01	4.82	5.26	2,787,062	341	12,687	53,034	39.6
one-stage_bin047	83.58	4.37	15.79	2,887,049	389	11,056	42,343	69.9
one-stage_bin048	97.72	0.55	0	3,374,143	299	23,305	86,113	37.6
one-stage_bin049	92.62	1.09	0	2,914,375	84	56,532	252,196	44.9
one-stage_bin050	68.47	3.37	100	2,302,562	109	41,825	108,437	50.3
one-stage_bin051	94.27	0.56	0	3,379,328	21	272,288	661,789	42.2
one-stage_bin052	51.57	2.38	75	1,807,325	253	9,930	32,965	49.3
one-stage_bin053	88.41	1.11	66.67	2,439,451	344	9,682	38,722	33.9
one-stage_bin054	89.09	6	0	1,910,147	157	18,631	75,280	55.9
one-stage_bin055	84.99	66.67	2.78	4,907,584	1,408	3,522	16,691	53.8
one-stage_bin056	79.22	5.14	0	2,647,480	662	4,311	16,040	60.8
one-stage_bin057	91.2	1.85	0	4,475,216	215	34,083	130,761	48.1
one-stage_bin058	79.78	32.71	1.27	3,695,288	729	6,228	33,798	60.9
one-stage_bin059	97.29	1.23	0	2,384,460	104	38,493	149,789	30.7
one-stage_bin060	53.89	15.33	0	3,000,535	890	3,310	15,708	56.2
one-stage_bin061	49.24	8.08	0	1,815,997	524	3,432	16,748	32.3
one-stage_bin062	85.82	33.36	0	3,609,164	424	17,507	84,166	49.6
one-stage_bin063	95.43	0.54	0	2,817,186	50	113,143	278,422	37.6
one-stage_bin064	76.83	7.06	16.67	2,589,269	143	83,474	277,793	58.2
one-stage_bin065	55.88	0	0	581,059	72	13,767	35,025	33.9
one-stage_bin066	94.41	34.48	1.89	2,881,991	677	4,668	23,213	38.6

one-stage_bin067	38.42	1.49	0	601,728	128	5,051	21,495	35.2
one-stage_bin068	73.98	0	0	830,943	18	73,974	214,329	37.2
one-stage_bin069	99.01	0.49	100	3,150,113	38	158,910	276,331	33.4
one-stage_bin070	0	0	0	236,704	81	2,670	8,493	67.1
one-stage_bin071	56.97	0.47	0	413,255	71	7,187	18,047	34.2
one-stage_bin072	97.27	0	0	3,858,112	67	107,716	342,130	48.5
one-stage_bin073	93.7	5.04	0	3,203,705	137	46,289	150,228	41.2
one-stage_bin074	67.98	11.29	12.5	4,020,519	1,086	3,708	65,105	69.2
one-stage_bin075	63.54	11.85	9.09	2,183,986	661	3,288	21,391	62.3
one-stage_bin076	91.41	3.41	0	5,406,586	284	30,990	163,820	59.1
one-stage_bin077	94.12	6.9	13.33	2,240,761	400	7,493	40,747	48.5
one-stage_bin078	96.32	7.7	45.45	4,201,763	543	11,942	64,499	63.4
two-stage_bin001	98.56	1.65	0	3,239,341	7	2,384,790	2,384,790	42.3
two-stage_bin002	96.65	1.12	33.33	3,691,527	98	93,877	273,576	33.6
two-stage_bin003	95.45	0	0	4,201,651	9	1,703,990	1,986,035	69.4
two-stage_bin004	90.91	38.12	1.85	5,734,563	205	54,281	228,371	60.2
two-stage_bin005	87.19	0.51	50	3,311,770	176	47,006	207,938	69.1
two-stage_bin006	27.88	1.36	50	2,522,919	137	53,829	602,925	52.9
two-stage_bin007	95.57	1.14	33.33	3,339,125	40	240,285	460,669	66.5
two-stage_bin008	91.19	7.57	0	3,298,598	62	146,414	466,316	68
two-stage_bin009	89.43	38.08	21.78	4,826,693	409	27,653	132,634	69.8
two-stage_bin010	81.5	1.11	0	2,369,268	170	27,848	80,765	60.5
two-stage_bin011	95.73	1.76	0	3,139,755	7	2,954,805	2,954,805	56.3
two-stage_bin012	93.7	10.48	85.29	5,163,508	288	44,496	143,474	43.9
two-stage_bin013	97.44	2.14	0	2,845,922	69	126,369	380,907	73.7
two-stage_bin014	96.49	3.86	0	8,245,564	294	213,270	606,518	59.3
two-stage_bin015	95.45	14.56	3.7	5,702,479	483	27,145	125,909	66.6
two-stage_bin016	98.25	49.95	1.54	10,293,621	1,400	12,908	71,189	64.6
two-stage_bin017	78.76	9.11	27.27	4,429,720	180	83,198	274,017	57.5
two-stage_bin018	66.85	3.07	0	3,430,671	559	7,956	31,512	62.2
two-stage_bin019	33.7	5.17	0	2,346,053	635	3,753	24,085	63.7
two-stage_bin020	80.14	29.11	86.97	6,759,481	533	26,329	154,799	61.9
two-stage_bin021	47.2	4.27	0	2,787,805	741	4,008	15,746	72.5
two-stage_bin022	78.07	26.4	0	2,333,077	461	5,730	80,479	38.3
two-stage_bin023	98.77	6.17	0	3,135,179	182	45,698	383,427	29
two-stage_bin024	70.77	15.99	0	4,171,191	1,107	3,955	22,724	64.6
two-stage_bin025	64.4	1.12	0	1,156,386	44	137,312	306,243	44.9
two-stage_bin026	92.49	6.89	17.14	3,075,740	99	104,161	180,570	68.3
two-stage_bin027	92.89	1.85	42.86	3,446,903	181	41,915	133,337	34.9
two-stage_bin028	71.57	59.58	1.92	5,705,178	1,673	3,455	22,037	71.6
two-stage_bin029	98.25	71.05	3.95	5,875,066	916	16,180	162,498	69.3
two-stage_bin030	84.56	1.89	11.11	3,131,600	194	29,946	122,040	68.9
two-stage_bin031	59.65	0	0	7,116,325	339	52,124	269,808	62.2
two-stage_bin032	76.4	34.05	0	2,496,487	594	4,543	20,690	40.1
two-stage_bin033	92.4	11.33	7.69	4,692,649	948	6,201	49,666	60.3
two-stage_bin034	73.98	20.92	0	3,254,932	765	4,641	24,422	33.3
two-stage_bin035	100	9.48	0	3,862,158	72	196,672	458,356	42.6
two-stage_bin036	98.69	19.14	3.66	5,389,863	808	10,776	275,412	48.8
two-stage_bin037	89.86	2.87	40	2,227,921	394	7,351	32,899	33.6
two-stage_bin038	91.38	45.02	91.67	4,110,744	620	9,558	111,087	48.8
two-stage_bin039	49.31	8.36	0	2,610,985	745	3,522	13,480	55.4
two-stage_bin040	29.15	11.33	0	4,951,617	1,006	5,571	138,505	56.2
two-stage_bin041	72.53	1.1	83.33	2,357,971	438	6,504	31,340	67.6
two-stage_bin042	68.1	0	0	3,287,477	500	8,471	35,836	62.5
two-stage_bin043	93.42	40.6	81.82	6,324,270	591	61,425	458,208	53.4
two-stage_bin044	45.85	0	0	2,101,867	427	5,813	31,484	62.9
two-stage_bin045	39.84	7.57	0	4,649,941	1,142	4,144	82,553	62.6
two-stage_bin046	91.95	2.19	25	2,085,825	122	32,781	98,934	48.9
two-stage_bin047	55.64	11.01	0	2,196,236	620	3,591	33,023	66.5
two-stage_bin048	98.26	0.65	0	2,577,612	18	504,136	814,568	65.9
two-stage_bin049	88.86	6.68	6.67	3,805,348	485	12,383	42,744	37.5
two-stage_bin050	75.96	16.07	0	3,288,724	814	4,326	18,414	42
two-stage_bin051	60.07	8.13	5	2,560,668	704	3,694	39,717	70.3
two-stage_bin052	94.3	38.3	0.64	4,407,916	941	5,759	32,653	66.6
two-stage_bin053	39.51	2.26	0	1,290,828	371	3,530	12,994	64.4
two-stage_bin054	97.29	86.66	0.84	6,122,287	749	12,929	64,892	40.4

Table S3. Bin Taxonomy

Bin ID	Bin Taxonomy
one-stage_bin001	d__Bacteria; p__Planctomycetota; c__Brocadiae; o__Brocadiales; f__Brocadiaceae; g__Brocadia
one-stage_bin002	d__Bacteria; p__Proteobacteria; c__Gammaproteobacteria; o__Burkholderiales; f__Rhodocyclaceae; g__UTPRO2
one-stage_bin003	d__Bacteria; p__Proteobacteria; c__Gammaproteobacteria; o__Pseudomonadales; f__UBA5518; g__UBA5518
one-stage_bin004	d__Bacteria; p__Bacteroidota; c__Bacteroidia; o__AKYH767-A; f__OLB10; g__
one-stage_bin005	d__Bacteria; p__Chloroflexota; c__Anaerolineae; o__Anaerolineales; f__envOPS12; g__UBA7227
one-stage_bin006	d__Bacteria; p__Verrucomicrobiota; c__Verrucomicrobiae; o__Pedosphaerales; f__Pedosphaeraceae; g__
one-stage_bin007	d__Bacteria; p__Chloroflexota; c__Anaerolineae; o__Promineofilales; f__Promineofilaceae; g__Promineofilum
one-stage_bin008	d__Bacteria; p__Bacteroidota; c__Bacteroidia; o__Chitinophagales; f__Chitinophagaceae; g__UTBCD1
one-stage_bin009	d__Bacteria; p__Chloroflexota; c__Anaerolineae; o__Anaerolineales; f__envOPS12; g__OLB14
one-stage_bin010	d__Bacteria; p__Proteobacteria; c__Gammaproteobacteria
one-stage_bin011	d__Bacteria; p__Proteobacteria; c__Gammaproteobacteria; o__Xanthomonadales; f__Rhodanobacteraceae; g__Dokdonella
one-stage_bin012	d__Bacteria; p__Proteobacteria; c__Gammaproteobacteria; o__GCA-2729495; f__GCA-2729495; g__
one-stage_bin013	d__Bacteria; p__Proteobacteria; c__Gammaproteobacteria; o__Burkholderiales; f__Nitrosomonadaceae; g__Nitrosomonas
one-stage_bin014	d__Bacteria; p__Acidobacteriota; c__Acidobacteriae; o__Bryobacterales; f__Bryobacteraceae; g__
one-stage_bin015	d__Bacteria; p__Chloroflexota; c__Anaerolineae; o__Anaerolineales; f__envOPS12; g__OLB14
one-stage_bin016	d__Bacteria; p__Proteobacteria; c__Gammaproteobacteria; o__Burkholderiales; f__Burkholderiaceae; g__SCN-69-89
one-stage_bin017	d__Bacteria; p__Binatota; c__Binatia; o__UTPRO1; f__UTPRO1; g__UTPRO1
one-stage_bin018	d__Bacteria; p__Acidobacteriota; c__Vicinamibacteria; o__Vicinamibacterales; f__UBA2999; g__
one-stage_bin019	d__Bacteria; p__Proteobacteria; c__Gammaproteobacteria; o__Burkholderiales; f__Palsa-1005; g__
one-stage_bin020	d__Bacteria; p__Proteobacteria; c__Gammaproteobacteria; o__Burkholderiales; f__Burkholderiaceae; g__Rubrivivax
one-stage_bin021	d__Bacteria; p__Bacteroidota; c__Bacteroidia; o__AKYH767; f__UBA4408; g__
one-stage_bin022	d__Bacteria; p__Chloroflexota; c__Anaerolineae; o__Anaerolineales; f__envOPS12; g__UBA12294
one-stage_bin023	d__Bacteria; p__Chloroflexota; c__Anaerolineae; o__Anaerolineales; f__Anaerolineaceae; g__UBA6170
one-stage_bin024	d__Bacteria; p__Proteobacteria; c__Gammaproteobacteria; o__Steroidobacterales; f__Steroidobacteraceae; g__UBA964
one-stage_bin025	d__Bacteria; p__Chloroflexota; c__Anaerolineae; o__Anaerolineales; f__Anaerolineaceae; g__
one-stage_bin026	d__Bacteria; p__Acidobacteriota; c__Acidobacteriae; o__Bryobacterales; f__; g__
one-stage_bin027	d__Bacteria; p__Acidobacteriota; c__Acidobacteriae; o__Bryobacterales; f__Bryobacteraceae; g__
one-stage_bin028	d__Bacteria; p__Chloroflexota; c__Dehalococcoidia; o__UBA2991; f__UBA2991; g__
one-stage_bin029	d__Bacteria; p__Actinobacteriota; c__Thermoleophilia; o__20CM-4-69-9; f__; g__
one-stage_bin030	d__Bacteria; p__Planctomycetota; c__UBA8742; o__UBA2392; f__UBA2392; g__UBA2392
one-stage_bin031	d__Bacteria; p__Proteobacteria; c__Gammaproteobacteria; o__Burkholderiales; f__Burkholderiaceae; g__Rubrivivax
one-stage_bin032	d__Archaea; p__Euryarchaeota
one-stage_bin033	d__Bacteria; p__Chloroflexota; c__Anaerolineae; o__Anaerolineales; f__UBA4823; g__UTCFX2
one-stage_bin034	d__Bacteria; p__Proteobacteria; c__Gammaproteobacteria; o__Xanthomonadales; f__; g__
one-stage_bin035	d__Bacteria; p__Bacteroidota; c__Bacteroidia; o__Chitinophagales; f__Saprospiraceae; g__OLB9
one-stage_bin036	d__Bacteria; p__Myxococcota; c__Polyangia; o__Polyangiales; f__Polyangiaceae; g__
one-stage_bin037	d__Bacteria; p__Proteobacteria; c__Gammaproteobacteria; o__Pseudomonadales; f__UBA7239; g__UBA7239
one-stage_bin038	d__Bacteria; p__Proteobacteria; c__Gammaproteobacteria; o__Burkholderiales; f__Nitrosomonadaceae; g__Nitrosomonas
one-stage_bin039	d__Bacteria; p__Proteobacteria; c__Gammaproteobacteria; o__; f__; g__
one-stage_bin040	d__Bacteria; p__Planctomycetota; c__Phycisphaerae; o__Phycisphaerales; f__Phycisphaeraceae; g__
one-stage_bin041	d__Bacteria; p__Proteobacteria; c__Gammaproteobacteria; o__Xanthomonadales; f__; g__
one-stage_bin042	d__Bacteria; p__Actinobacteriota; c__Acidimicrobiia; o__Microtrichales; f__Microtrichaceae; g__IMCC26207
one-stage_bin043	d__Bacteria; p__Myxococcota; c__Polyangia; o__Polyangiales; f__Polyangiaceae; g__
one-stage_bin044	d__Bacteria; p__Myxococcota; c__Polyangia; o__Polyangiales; f__Polyangiaceae; g__

one-stage_bin045	d__Bacteria; p__Bacteroidota; c__Bacteroidia; o__; f__; g__
one-stage_bin046	d__Bacteria; p__Bacteroidota; c__Bacteroidia; o__Flavobacteriales; f__; g__
one-stage_bin047	d__Bacteria; p__Proteobacteria; c__Gammaproteobacteria; o__Burkholderiales; f__Burkholderiaceae; g__Ottowia
one-stage_bin048	d__Bacteria; p__Bacteroidota; c__Bacteroidia; o__NS11-12g; f__UBA955; g__UBA6161
one-stage_bin049	d__Bacteria; p__Bacteroidota; c__Kapabacteria; o__Kapabacteriales; f__UBA961; g__
one-stage_bin050	d__Bacteria; p__Spirochaetota; c__Leptospirae; o__Turneriellales; f__Turneriellaceae; g__Turneriella
one-stage_bin051	d__Bacteria; p__Bacteroidota; c__Ignavibacteria; o__Ignavibacteriales; f__Ignavibacteriaceae_A; g__UTCHB3
one-stage_bin052	d__Bacteria; p__Spirochaetota; c__Leptospirae; o__Turneriellales; f__Turneriellaceae; g__Turneriella
one-stage_bin053	d__Bacteria; p__Bacteroidota; c__Bacteroidia; o__NS11-12g; f__UKL13-3; g__UBA6183
one-stage_bin054	d__Bacteria; p__Chloroflexota; c__Anaerolineae; o__Anaerolineales; f__Anaerolineaceae; g__49-20
one-stage_bin055	d__Bacteria; p__Bdellovibrionota; c__Bacteriovoracia; o__; f__; g__
one-stage_bin056	d__Bacteria; p__Gemmatimonadota; c__Gemmatimonadetes; o__Gemmatimonadales; f__Gemmatimonadaceae; g__
one-stage_bin057	d__Bacteria; p__Eremiobacterota; c__UBP9; o__UBA4705; f__; g__
one-stage_bin058	d__Bacteria; p__Verrucomicrobiota; c__Verrucomicrobiae; o__Opitutales; f__Opitutaceae; g__Cephaloticoccus
one-stage_bin059	d__Bacteria; p__Bacteroidota; c__Bacteroidia; o__Chitinophagales; f__Chitinophagaceae; g__UBA1930
one-stage_bin060	d__Bacteria; p__Verrucomicrobiota; c__Verrucomicrobiae; o__; f__; g__
one-stage_bin061	d__Bacteria; p__Bacteroidota; c__Bacteroidia; o__Chitinophagales; f__Chitinophagaceae; g__OLB11
one-stage_bin062	d__Bacteria; p__Chloroflexota; c__Anaerolineae; o__Anaerolineales; f__Anaerolineaceae; g__Brevefilum
one-stage_bin063	d__Bacteria; p__Bacteroidota; c__Bacteroidia; o__Bacteroidales; f__4484-276; g__
one-stage_bin064	d__Bacteria; p__Firmicutes_A; c__Clostridia; o__Christensenellales; f__CAG-74; g__DTU024
one-stage_bin065	d__Bacteria; p__Patescibacteria; c__Dojkabacteria; o__SC72; f__SC72; g__UBA5232
one-stage_bin066	d__Bacteria; p__Cloacimonadota; c__Cloacimonadia; o__Cloacimonadales; f__Cloacimonadaceae; g__Cloacimonas
one-stage_bin067	unclassified
one-stage_bin068	d__Bacteria; p__Patescibacteria; c__Microgenomatia; o__Shapirobacterales; f__UBA12405; g__UBA1435
one-stage_bin069	d__Bacteria; p__Bacteroidota; c__Bacteroidia; o__Chitinophagales; f__; g__
one-stage_bin070	d__Bacteria; p__Patescibacteria; c__Dojkabacteria; o__SC72; f__SC72; g__UBA2177
one-stage_bin071	d__Bacteria; p__Bacteroidota; c__Bacteroidia; o__; f__; g__
one-stage_bin072	d__Bacteria; p__Desulfobacterota; c__Syntrophorhabdia; o__Syntrophorhabdales; f__Syntrophorhabdaceae; g__
one-stage_bin073	d__Bacteria; p__Armatimonadota; c__UBA10988; o__UBA10988; f__; g__
one-stage_bin074	d__Bacteria; p__Verrucomicrobiota; c__Verrucomicrobiae; o__Chthoniobacterales; f__Terrimicrobiaceae; g__
one-stage_bin075	d__Bacteria; p__Planctomycetota; c__Planctomycetes; o__Gemmatales; f__Gemmataceae; g__Fimbriiglobus
one-stage_bin076	d__Bacteria; p__Bacteroidota; c__Bacteroidia; o__Bacteroidales; f__UBA932; g__DMER64
one-stage_bin077	d__Bacteria; p__Proteobacteria; c__Gammaproteobacteria; o__Burkholderiales; f__Burkholderiaceae; g__Candidimonas
one-stage_bin078	d__Bacteria; p__Proteobacteria; c__Gammaproteobacteria; o__Steroidobacterales; f__Steroidobacteraceae; g__UBA964
two-stage_bin001	d__Bacteria; p__Planctomycetota; c__Brocadiae; o__Brocadiales; f__Brocadiaceae; g__Brocadia
two-stage_bin002	d__Bacteria; p__Bacteroidota; c__Ignavibacteria; o__Ignavibacteriales; f__Ignavibacteriaceae; g__Ignavibacterium
two-stage_bin003	d__Bacteria; p__Planctomycetota; c__Phycisphaerae; o__UBA1845; f__Fen-1342; g__
two-stage_bin004	d__Bacteria; p__Chloroflexota; c__Anaerolineae; o__Anaerolineales; f__envOPS12; g__UBA7227
two-stage_bin005	d__Bacteria; p__Proteobacteria; c__Gammaproteobacteria; o__Burkholderiales; f__Burkholderiaceae; g__SCN-69-89
two-stage_bin006	d__Bacteria; p__Chloroflexota; c__Anaerolineae; o__Anaerolineales; f__UBA4823; g__UTCFX2
two-stage_bin007	d__Bacteria; p__Proteobacteria; c__Gammaproteobacteria; o__Burkholderiales; f__Rhodocyclaceae; g__UTPRO2
two-stage_bin008	d__Bacteria; p__Proteobacteria; c__Gammaproteobacteria; o__GCA-2729495; f__GCA-2729495; g__
two-stage_bin009	d__Bacteria; p__Proteobacteria; c__Gammaproteobacteria; o__Xanthomonadales; f__Rhodanobacteraceae; g__Dokdonella
two-stage_bin010	d__Bacteria; p__Proteobacteria; c__Gammaproteobacteria; o__Burkholderiales; f__Burkholderiaceae; g__Comamonas
two-stage_bin011	d__Bacteria; p__Acidobacteriota; c__Blastocatellia; o__Pyrinomonadales; f__Pyrinomonadaceae; g__OLB17
two-stage_bin012	d__Bacteria; p__Proteobacteria; c__Gammaproteobacteria; o__Enterobacterales; f__Shewanellaceae; g__Shewanella
two-stage_bin013	d__Bacteria; p__Actinobacteriota; c__Acidimicrobiia; o__IMCC26256; f__; g__
two-stage_bin014	d__Bacteria; p__Acidobacteriota; c__Acidobacteriae; o__Bryobacterales; f__Bryobacteraceae; g__
two-stage_bin015	d__Bacteria; p__Chloroflexota; c__Anaerolineae; o__SBR1031; f__UBA2029; g__
two-stage_bin016	d__Bacteria; p__Planctomycetota; c__Planctomycetes; o__Pirellulales; f__Thermoguttaceae; g__

two-stage_bin017	d__Bacteria; p__Proteobacteria; c__Gammaproteobacteria; o__Pseudomonadales; f__Pseudomonadaceae; g__Pseudomonas_E
two-stage_bin018	d__Bacteria; p__Acidobacteriota; c__Acidobacteriae; o__Bryobacterales; f__Bryobacteraceae; g__
two-stage_bin019	d__Bacteria; p__Verrucomicrobiota; c__Verrucomicrobiae; o__; f__; g__
two-stage_bin020	d__Bacteria; p__Proteobacteria; c__Gammaproteobacteria; o__Pseudomonadales; f__Pseudomonadaceae; g__Pseudomonas_E
two-stage_bin021	d__Bacteria; p__Acidobacteriota; c__Thermoanaerobaculia; o__UBA5066; f__UBA5066; g__UBA5066
two-stage_bin022	d__Bacteria; p__Patescibacteria; c__Doudnabacteria; o__UBA920; f__UBA920; g__UBA920
two-stage_bin023	d__Bacteria; p__Bacteroidota; c__Bacteroidia; o__Chitinophagales; f__Chitinophagaceae; g__UBA1930
two-stage_bin024	d__Bacteria; p__Chloroflexota; c__Anaerolineae; o__Caldilineales; f__Caldilineaceae; g__
two-stage_bin025	d__Bacteria; p__Patescibacteria; c__Microgenomatia; o__UBA1400; f__UBA12108; g__UBA12108
two-stage_bin026	d__Bacteria; p__Proteobacteria; c__Gammaproteobacteria; o__Burkholderiales; f__Burkholderiaceae; g__Comamonas_C
two-stage_bin027	d__Bacteria; p__Bacteroidota; c__Bacteroidia; o__Flavobacteriales; f__Weeksellaceae; g__Chryseobacterium_A
two-stage_bin028	d__Bacteria; p__Acidobacteriota; c__Vicinamibacteria; o__; f__; g__
two-stage_bin029	d__Bacteria; p__Chloroflexota; c__Dehalococcoidia; o__UBA2979; f__UBA2979; g__
two-stage_bin030	d__Bacteria; p__Proteobacteria; c__Gammaproteobacteria; o__Burkholderiales; f__Burkholderiaceae; g__Comamonas_D
two-stage_bin031	d__Bacteria; p__Proteobacteria; c__Gammaproteobacteria; o__Pseudomonadales; f__Pseudomonadaceae; g__Pseudomonas_E
two-stage_bin032	d__Bacteria; p__Bdellovibrionota; c__Bdellovibrionia; o__; f__; g__
two-stage_bin033	d__Bacteria; p__Verrucomicrobiota; c__Kiritimatiellae; o__; f__; g__
two-stage_bin034	d__Bacteria; p__Bacteroidota; c__Bacteroidia; o__Flavobacteriales; f__koll-22; g__
two-stage_bin035	d__Bacteria; p__Bacteroidota; c__Bacteroidia; o__Chitinophagales; f__Chitinophagaceae; g__
two-stage_bin036	d__Bacteria; p__Proteobacteria; c__Gammaproteobacteria; o__Burkholderiales; f__Nitrosomonadaceae; g__Nitrosomonas
two-stage_bin037	d__Bacteria; p__Firmicutes_A; c__Clostridia; o__Peptostreptococcales; f__Filifactoraceae; g__Acetoanaerobium
two-stage_bin038	d__Bacteria; p__Proteobacteria; c__Gammaproteobacteria; o__Pseudomonadales; f__Pseudomonadaceae; g__Pseudomonas_C
two-stage_bin039	d__Bacteria; p__Desulfobacterota; c__Syntrophia; o__Syntrophales; f__Smithellaceae; g__UBA8904
two-stage_bin040	d__Bacteria; p__Proteobacteria; c__Gammaproteobacteria; o__Enterobacterales; f__Aeromonadaceae; g__Aeromonas
two-stage_bin041	d__Bacteria; p__Proteobacteria; c__Alphaproteobacteria; o__Sphingomonadales; f__Sphingomonadaceae; g__Sphingopyxis
two-stage_bin042	d__Bacteria; p__Proteobacteria; c__Gammaproteobacteria; o__Enterobacterales; f__Aeromonadaceae; g__Aeromonas
two-stage_bin043	d__Bacteria; p__Proteobacteria; c__Gammaproteobacteria; o__Enterobacterales; f__Enterobacteriaceae; g__Lelliottia
two-stage_bin044	d__Bacteria; p__Proteobacteria; c__Gammaproteobacteria; o__Burkholderiales; f__Burkholderiaceae; g__Comamonas
two-stage_bin045	d__Bacteria; p__Proteobacteria; c__Gammaproteobacteria; o__Pseudomonadales; f__Pseudomonadaceae; g__Pseudomonas_A
two-stage_bin046	d__Bacteria; p__Bacteroidota; c__Bacteroidia; o__Bacteroidales; f__UBA932; g__DMER64
two-stage_bin047	d__Bacteria; p__Proteobacteria; c__Alphaproteobacteria; o__Rhizobiales; f__Rhizobiaceae; g__Aquamicrobium
two-stage_bin048	d__Bacteria; p__Proteobacteria; c__Alphaproteobacteria; o__Sphingomonadales; f__Sphingomonadaceae; g__
two-stage_bin049	d__Bacteria; p__Bacteroidota; c__Bacteroidia; o__Chitinophagales; f__Saprospiraceae; g__OLB9
two-stage_bin050	d__Bacteria; p__Desulfobacterota; c__Syntrophorhabdia; o__Syntrophorhabdales; f__Syntrophorhabdaceae; g__
two-stage_bin051	d__Bacteria; p__Proteobacteria; c__Alphaproteobacteria; o__Rhodobacterales; f__Rhodobacteraceae; g__UBA996
two-stage_bin052	d__Bacteria; p__Proteobacteria; c__Alphaproteobacteria; o__Sphingomonadales; f__Sphingomonadaceae; g__UBA6171
two-stage_bin053	d__Bacteria; p__Desulfobacterota; c__Syntrophia; o__Syntrophales; f__UBA2192; g__UBA2192
two-stage_bin054	d__Bacteria; p__Bacteroidota; c__Bacteroidia; o__Chitinophagales; f__Chitinophagaceae; g__

Table S4. Bin Annotations

BinID	Predicted CDS	# EggNOG hits	% Annotated	Nitrogen Transformation Genes																CAZy Annotations					
				napA	*napB*	*nrfA*	*nrfH*	*narG*	*narH*	*narI*	*nirB*	*nirD*	*nirK*	*nirS*	*norB*	*norC*	*nosZ*	*amoA*	*hao*	AA	CBM	CE	GH	GT	PL
one-stage_bin001	2728	2379	87.21%			1	1	1	3		1								2	2	4	5	17	84	0
one-stage_bin002	2952	2716	92.01%	1	1			1	1	1				2			1		1	6	0	2	13	31	1
one-stage_bin003	4156	3808	91.63%					1	4	1				1			1			13	1	7	19	41	2
one-stage_bin004	2271	2063	90.84%					1	1	1					1		1			1	8	6	10	52	1
one-stage_bin005	3093	2581	83.45%			1	1	2	2	2				1						3	7	16	26	57	10
one-stage_bin006	3727	2976	79.85%						1				1							5	13	18	96	43	4
one-stage_bin007	3945	3045	77.19%			1	1										1			5	10	11	34	90	9
one-stage_bin008	3085	2693	87.29%					1	1	1							1			4	7	21	51	43	6
one-stage_bin009	2514	2158	85.84%																	4	3	7	19	49	5
one-stage_bin010	4019	3376	84.00%					3	5	2				1					1	9	5	20	13	29	1
one-stage_bin011	3217	2895	89.99%					1	1	1			1	1	1	1				7	1	15	14	39	9
one-stage_bin012	5598	5259	93.94%					1	3	1	1			2	2		1			18	2	14	26	52	5
one-stage_bin013	2089	1997	95.60%												1	1		1	1	8	0	4	15	27	2
one-stage_bin014	6184	5273	85.27%					4	3	3					1		1		1	13	3	53	130	99	22
one-stage_bin015	3151	2644	83.91%					3	2	2										3	6	16	22	68	9
one-stage_bin016	2786	2602	93.40%							1				1			1			2	0	4	18	26	1
one-stage_bin017	3296	2768	83.98%						2				2				2			7	3	13	24	79	4
one-stage_bin018	5783	4668	80.72%					4	3	4	1			2	1	1	4			13	13	22	28	105	5
one-stage_bin019	2808	2503	89.14%					1	1	1				1						7	0	11	9	31	0
one-stage_bin020	2919	2727	93.42%					1	1	2				2					1	7	0	10	14	26	1
one-stage_bin021	2978	2595	87.14%					1	1	1					1		1			2	3	16	16	57	1
one-stage_bin022	2428	2040	84.02%					4	2	2			1							2	5	9	14	36	4
one-stage_bin023	6702	5356	79.92%											2			2			6	11	26	90	77	4
one-stage_bin024	3033	2689	88.66%										1						1	10	3	12	12	38	1
one-stage_bin025	4326	2846	65.79%			3	2	6	3	2					2					3	4	8	49	38	3
one-stage_bin026	5704	4524	79.31%			1	1	1	3	1					1		1			8	3	40	96	71	9
one-stage_bin027	4727	3846	81.36%			2	1								3					10	4	26	106	63	8
one-stage_bin028	4521	3354	74.19%					1	4								1			8	3	17	20	26	1
one-stage_bin029	4044	3136	77.55%												1					3	4	10	20	43	3
one-stage_bin030	3862	2594	67.17%			1	1	1	1	1		1		1	1					3	2	8	5	51	10
one-stage_bin031	3676	3434	93.42%					2						3					1	11	0	11	10	29	2
one-stage_bin032	5170	4485	86.75%					2	1	1			1				1			6	6	14	25	62	5
one-stage_bin033	2217	1555	70.14%					3	1	1										2	4	4	36	22	3
one-stage_bin034	6741	5820	86.34%					4	1	1			1	3	1	1	1			13	0	14	25	63	5
one-stage_bin035	2942	2491	84.67%												1	1	1			4	3	15	18	43	4
one-stage_bin036	4999	3517	70.35%	1	1	1		1	2	1			2		1	1	1			25	3	15	29	46	3
one-stage_bin037	2267	2077	91.62%					1	1	1										7	1	5	9	36	1
one-stage_bin038	2200	2062	93.73%												2	1		1	3	11	0	4	18	20	2
one-stage_bin039	5680	4899	86.25%			1			5	3				1						11	3	13	26	80	3
one-stage_bin040	5476	4162	76.00%			1			1	1			1		1		4			4	7	17	106	61	19
one-stage_bin041	4154	3362	80.93%					3	2	1				1			1			2	0	5	16	25	2
one-stage_bin042	3455	2967	85.88%						1				3				1			12	6	28	30	55	1
one-stage_bin043	3931	2963	75.38%	1	1			1	1	1				1	1	1	1			13	2	18	23	38	9
one-stage_bin044	5228	3813	72.93%	2	1			1	1	1			1				1			11	4	21	31	56	3

one-stage_bin045	2711	2320	85.58%						1				1		1		2			6	9	16	8	48	2
one-stage_bin046	2624	2194	83.61%					2	1	1			2		1		1			6	4	11	12	56	2
one-stage_bin047	2985	2815	94.30%								3	1		1	1	1				10	0	5	10	22	1
one-stage_bin048	2994	2460	82.16%					1	1	1					1		1			3	8	8	5	44	0
one-stage_bin049	2290	1880	82.10%					1	1	1			1				1			1	1	7	30	50	4
one-stage_bin050	2216	1549	69.90%										1	1						3	0	5	4	14	1
one-stage_bin051	2600	2223	85.50%			1	1										1			1	4	13	50	49	2
one-stage_bin052	1912	1306	68.31%																	3	1	5	8	22	0
one-stage_bin053	2122	1871	88.17%										2		1		2			2	6	9	9	46	2
one-stage_bin054	1755	1523	86.78%																	1	4	7	22	38	0
one-stage_bin055	5829	4324	74.18%						2				1				2			5	5	19	46	68	11
one-stage_bin056	3047	2355	77.29%										1				2			3	1	14	17	24	5
one-stage_bin057	4093	2719	66.43%						2				1				1			3	1	19	29	72	1
one-stage_bin058	3847	3111	80.87%										2							3	5	15	87	56	9
one-stage_bin059	2106	1878	89.17%					1	1	1							1			1	5	11	10	34	1
one-stage_bin060	3643	2534	69.56%										1				1			5	1	5	48	38	3
one-stage_bin061	1922	1446	75.23%										1							2	1	1	6	27	1
one-stage_bin062	3597	2906	80.79%																	0	2	14	41	44	1
one-stage_bin063	2189	1961	89.58%			1	1													2	8	5	32	36	2
one-stage_bin064	2462	2063	83.79%																	1	4	18	59	14	0
one-stage_bin065	578	379	65.57%																	0	1	2	1	15	0
one-stage_bin066	2903	2471	85.12%																	2	4	20	53	35	0
one-stage_bin067	668	489	73.20%																	2	0	2	1	6	0
one-stage_bin068	861	571	66.32%																	0	2	0	2	42	0
one-stage_bin069	2700	2348	86.96%										1		1	1	1			5	3	12	16	40	1
one-stage_bin070	268	197	73.51%																	0	1	1	5	3	0
one-stage_bin071	435	289	66.44%																	0	0	1	1	8	0
one-stage_bin072	2969	2467	83.09%										1		1	1	1			3	4	12	13	86	2
one-stage_bin073	3042	2528	83.10%																	6	1	3	12	48	1
one-stage_bin074	4306	3209	74.52%					1	1	2					1					4	8	23	81	62	8
one-stage_bin075	2556	2033	79.54%			1		1									1			3	5	16	37	36	1
one-stage_bin076	4539	3721	81.98%												1					4	5	34	29	73	2
one-stage_bin077	2379	2054	86.34%																	0	3	16	76	21	1
one-stage_bin078	4263	4067	95.40%					1	2	1			3		2					9	0	12	21	36	2
two-stage_bin001	2715	2354	86.70%			1	1	1	3		1								2	2	3	5	17	85	0
two-stage_bin002	3140	2709	86.27%			1	1	1	1	1							1			3	3	18	34	60	6
two-stage_bin003	3416	2551	74.68%			1	1		1								1			1	5	15	27	68	5
two-stage_bin004	5197	4206	80.93%			1	1	3	3	3				1						4	8	25	38	112	8
two-stage_bin005	3290	2961	90.00%					1	1					3			1		1	8	0	4	19	35	1
two-stage_bin006	2428	1597	65.77%			1	1													1	4	6	15	56	3
two-stage_bin007	3354	3090	92.13%	1	1			1	1	1				2	1		1		1	6	0	2	17	27	1
two-stage_bin008	3014	2695	89.42%					1	2					2	2					8	2	12	15	49	2
two-stage_bin009	4578	3912	85.45%					2	3	2			1	1	1	1				7	1	11	17	55	6
two-stage_bin010	2197	2036	92.67%											1	2	2	1			3	0	4	10	13	1
two-stage_bin011	2795	2226	79.64%					1	1	1							1			2	3	15	28	58	2
two-stage_bin012	4588	4245	92.52%	2	2	2		1	1	1					2	1				3	4	12	10	20	3
two-stage_bin013	2645	2268	85.75%					1	4	1			2							8	0	21	26	38	0
two-stage_bin014	7542	5950	78.89%			1	1	2	2	2										13	10	67	185	129	28
two-stage_bin015	5421	4384	80.87%					1	2	1									3	4	11	22	80	96	7
two-stage_bin016	9461	7436	78.60%			2	2		1						1		1			13	15	57	320	112	31

two-stage_bin017	4294	3715	86.52%					2	1	1	1	1	1		1	1	1			9	2	4	11	26	1
two-stage_bin018	3449	2936	85.13%					4	2	1					1				1	6	3	34	77	55	8
two-stage_bin019	2626	2054	78.22%												1					4	2	13	66	30	7
two-stage_bin020	6371	6067	95.23%						1	1	2	1		1	1	1	1			11	0	9	35	39	4
two-stage_bin021	3047	2360	77.45%			1	1	1												4	2	8	24	47	6
two-stage_bin022	2707	1972	72.85%										1				1			2	8	6	7	27	1
two-stage_bin023	3239	2315	71.47%										1				3			2	5	10	11	62	1
two-stage_bin024	4988	3731	74.80%			3	1	2	2			1	2	3	1					5	4	18	46	59	3
two-stage_bin025	1330	815	61.28%																	0	2	0	2	40	2
two-stage_bin026	3012	2888	95.88%												1	1			1	8	0	5	13	18	1
two-stage_bin027	3209	2907	90.59%										1				1			4	2	13	20	57	1
two-stage_bin028	6934	5471	78.90%							1									1	9	6	20	36	111	3
two-stage_bin029	6566	4903	74.67%					3	3	2			2				1			14	8	22	25	34	2
two-stage_bin030	3032	2918	96.24%					1	1	1	1	1		1	1					7	0	5	6	13	1
two-stage_bin031	6584	6181	93.88%					2	1	1	2	1		2	1	1	2			13	1	6	33	57	1
two-stage_bin032	3269	2133	65.25%						1		1		1				1			4	1	4	11	32	1
two-stage_bin033	5420	3947	72.82%			2	1				1		1							4	3	13	54	80	2
two-stage_bin034	4005	3098	77.35%					1	1	1				2						1	5	8	9	77	0
two-stage_bin035	3448	2843	82.45%										1				1			5	4	21	54	43	5
two-stage_bin036	6160	4745	77.03%												1	1		1	2	12	1	9	26	56	2
two-stage_bin037	2709	2362	87.19%																	0	0	2	12	16	0
two-stage_bin038	4167	3917	94.00%								1	1		1	5	3	1			8	2	5	14	29	1
two-stage_bin039	3572	2510	70.27%					3					1							5	2	7	30	29	1
two-stage_bin040	6243	4181	66.97%	1	1										1		2			5	0	2	22	27	4
two-stage_bin041	2528	2370	93.75%					2	1	1					1					3	1	12	11	9	0
two-stage_bin042	3474	3223	92.77%	2	1	1					2	1					1			2	2	4	39	21	1
two-stage_bin043	6320	6135	97.07%					6	4	5	3	1								6	1	17	82	50	7
two-stage_bin044	2335	2147	91.95%					1									1			4	0	4	6	19	1
two-stage_bin045	5684	4990	87.79%								3			2	4		2			15	1	5	23	24	1
two-stage_bin046	1915	1713	89.45%																	0	2	21	72	20	1
two-stage_bin047	2735	2497	91.30%					1	1	1										6	0	4	16	18	0
two-stage_bin048	2365	2221	93.91%												1	1				9	0	3	8	10	1
two-stage_bin049	3842	3005	78.21%														2			4	2	20	39	50	5
two-stage_bin050	4410	3308	75.01%										1							7	1	4	22	37	1
two-stage_bin051	2928	2575	87.94%					1		1										3	0	7	14	36	1
two-stage_bin052	5033	4633	92.05%						3	3				2	1	1			3	15	1	13	26	30	2
two-stage_bin053	1596	1291	80.89%																1	0	1	2	13	12	0
two-stage_bin054	6245	5588	89.48%					1					2				3			10	5	33	107	91	6

Table S5: Contingency table of summed RPKM values for binned contigs containing either a nir (nirK or niS) or nrfH gene and chi-square test

	nirK and nirS	nrfH
One-stage granules	142.3	13.01
Two-stage granules	85.98	101.2

$X^2 = 79.72$, $p = 4.3E-19$
